# GPI-anchor signal sequence influences PrP^C^ sorting, shedding and signalling, and impacts on different pathomechanistic aspects of prion disease in mice

**DOI:** 10.1371/journal.ppat.1007520

**Published:** 2019-01-04

**Authors:** Berta Puig, Hermann C. Altmeppen, Luise Linsenmeier, Karima Chakroun, Florian Wegwitz, Ulrike K. Piontek, Jörg Tatzelt, Clive Bate, Tim Magnus, Markus Glatzel

**Affiliations:** 1 Institute of Neuropathology, University Medical Center Hamburg-Eppendorf, Hamburg, Germany; 2 Department of Neurology, Experimental Research in Stroke and Inflammation (ERSI), University Medical Center Hamburg-Eppendorf, Hamburg, Germany; 3 Department of General, Visceral and Pediatric Surgery, Göttingen Center for Molecular Biosciences, University Medical Center Göttingen, Götingen, Germany; 4 Adolf Butenandt Institute, Neurobiochemistry, Ludwig Maximilians University Munich, Munich, Germany; 5 Institute of Biochemistry and Pathobiochemistry, Biochemistry of Neurodegenerative Diseases, Ruhr University Bochum, Bochum, Germany; 6 Department of Pathology and Pathogen Biology, Royal Veterinary College, Hawkshead Lane, North Mymms, Herts, United Kingdom; Dartmouth College Geisel School of Medicine, UNITED STATES

## Abstract

The cellular prion protein (PrP^C^) is a cell surface glycoprotein attached to the membrane by a glycosylphosphatidylinositol (GPI)-anchor and plays a critical role in transmissible, neurodegenerative and fatal prion diseases. Alterations in membrane attachment influence PrP^C^-associated signaling, and the development of prion disease, yet our knowledge of the role of the GPI-anchor in localization, processing, and function of PrP^C^ *in vivo* is limited We exchanged the PrP^C^ GPI-anchor signal sequence of for that of Thy-1 (PrP^C^GPIThy-1) in cells and mice. We show that this modifies the GPI-anchor composition, which then lacks sialic acid, and that PrP^C^GPIThy-1 is preferentially localized in axons and is less prone to proteolytic shedding when compared to PrP^C^. Interestingly, after prion infection, mice expressing PrP^C^GPIThy-1 show a significant delay to terminal disease, a decrease of microglia/astrocyte activation, and altered MAPK signaling when compared to wild-type mice. Our results are the first to demonstrate *in vivo*, that the GPI-anchor signal sequence plays a fundamental role in the GPI-anchor composition, dictating the subcellular localization of a given protein and, in the case of PrP^C^, influencing the development of prion disease.

## Author summary

The prion protein (PrP^C^) is a glycoprotein attached to the neuronal surface via a GPI-anchor. When misfolded to PrP^Sc^, it leads to fatal neurodegenerative diseases which propagates from host to host. PrP^Sc^ is the principal component of the infectious agent of prion diseases, the “prion”. Misfolding occurs at the plasma membrane, and when PrP^C^ lacks the GPI-anchor, neuropathology and incubation time of prion disease are strongly modified. Moreover, the composition of the PrP^C^ GPI-anchor impacts on the conversion process. To study the role of the GPI-anchor in the pathophysiology of prion diseases *in vivo*, we have generated transgenic mice where the PrP^C^ GPI-signal sequence (GPI-SS) is replaced for the one of Thy-1, a neuronal protein with a distinct GPI-anchor and membrane localization. We found that the resulting protein, PrP^C^GPIThy-1, shows a different GPI-anchor composition, increased axonal localization, and reduced enzymatic shedding. After prion infection, disease progression is significantly delayed, and the neuropathology and cellular signaling are changed.

The present work demonstrates that the GPI-SS *per se* determines the GPI-anchor composition and localization of a given protein and it stresses the importance of PrP^C^ membrane anchorage in prion disease.

## Introduction

The cellular prion protein (PrP^C^) is a cell surface GPI-anchored protein (GPI-AP) with two putative N-glycosylation sites [1, 2] targeted to detergent-resistant membranes (DRMs; or lipid rafts)[3]. All GPI-APs share a common GPI-anchor core structure which is highly conserved among species and consists of ethanolamine phosphate linked through an amide group to the carboxyl terminus of the protein, three mannose residues, glucosamine, and a phosphatidylinositol (PI) group. During biosynthesis, the signal sequence for a GPI-anchor (GPI-SS) is rapidly removed after ER translocation, and a common GPI-anchor core is attached to the protein via a GPI-transamidase. Once attached to the protein, this core undergoes several modification steps during ER and Golgi transport involving both elimination/addition of side branching sugars to the glycan moiety together with fatty acid remodeling [4, 5]. How these side chains and lipid moieties are chosen, and how this, in turn, affects the trafficking of GPI-APs is unclear, but it is probably cell- and species-specific and may depend on the functional context [6]. It could also be, that the GPI-SS itself influences GPI-anchor remodeling [7] which could then affect its intracellular sorting [3, 4]. In polarized Madin-Darby canine kidney (MDCK) cells, N-glycosylated GPI-APs are mostly apically sorted, suggesting that the GPI-anchor is an apical sorting signal [8], yet PrP^C^ is an exception in this regard being basolaterally sorted in these cells [9, 10]. When the GPI-SS of PrP^C^ is replaced by the one of the DRM-resident Thy-1, PrP^C^ partially relocates from the basolateral to the apical side in MDCK cells [11]. Likewise, GPI-SS-dependent relocalization of EGFP-tagged proteins occurs in cultured cells [12].

Most recently, Bate *et al*. demonstrated that sialylation of the PrP^C^ GPI-anchor plays a role in its synaptic targeting [13]. Although it has already been shown that the GPI-SS influences intracellular sorting *in vitro*, how differences in the GPI-SS impact on the GPI-anchor composition in neuronal cells or in the brain, has not been investigated yet.

A key event in the pathogenesis of prion diseases is a (templated) conformational change of PrP^C^ to its misfolded isoform (PrP^Sc^), the critical component of prion infectivity [14]. Neuropathological characteristics of prion diseases include vacuolization of neuropil and white matter, astro- and microgliosis, neuronal loss and PrP^Sc^ deposition [15]. Of outstanding importance for the pathogenesis of prion diseases are the membrane attachment and lipid raft localization of PrP^C^ [16]. Thus, cells expressing GPI-anchorless PrP cannot be infected with prions [17], and cholesterol depletion or expression of a transmembrane PrP-CD4 fusion protein (shifting PrP-CD4 out of DRMs) in cells, interferes with prion propagation [18]. Furthermore, when cell membranes are treated with analogs of GPI-anchors, such as glucosamine-PI, the membrane composition is altered, and the formation of PrP^Sc^ is reduced, probably by displacing PrP^C^ from lipid rafts [19]. Along the same line, enzymatic elimination of one GPI-anchor acyl chain in cells removes PrP^C^ from DRMs and reduces PrP^Sc^ amounts [20]. Also, mice expressing GPI-anchorless PrP^C^ show delayed clinical onset of prion disease together with altered clinical and neuropathological presentation [21, 22].

Interestingly, the GPI-anchor of PrP^C^ bears a sialic acid, a rare modification for a mammalian GPI-AP [23]. PrP^C^ lacking sialic acid in its GPI-anchor cannot convert to PrP^Sc^ *in vitro* [13, 24]. Sialo and asialo GPI-anchored PrP^Sc^ are equally present in infected mice [25]. Therefore, the contribution of the GPI-anchor sialic acid modification to the conversion of PrP^C^ to PrP^Sc^ clearly needs further investigations.

In the present study and based on our previous results [11], we assessed how the substitution of the GPI-SS of PrP^C^ for that of Thy-1 influences the biology of the resulting chimeric PrP^C^GPIThy-1 *in vivo*, and how this impacts on the pathophysiology of prion disease. We provide evidence that this replacement alters the resulting GPI-anchor composition regarding sialic acid content and leads to relocalization of PrP^C^GPIThy-1, increasing its presence in axons when compared to wild-type PrP^C^ (WTPrP^C^). This is accompanied by decreased proteolytic shedding of the protein. After intracerebral challenge with mouse-adapted prions, incubation time to clinical prion disease is extended, correlating with decreased activation of the mitogen-activated protein kinase ERK and reduced glial activation.

## Results

### Characterization of transgenic PrP^C^GPIThy-1 mice

**Fig 1:**
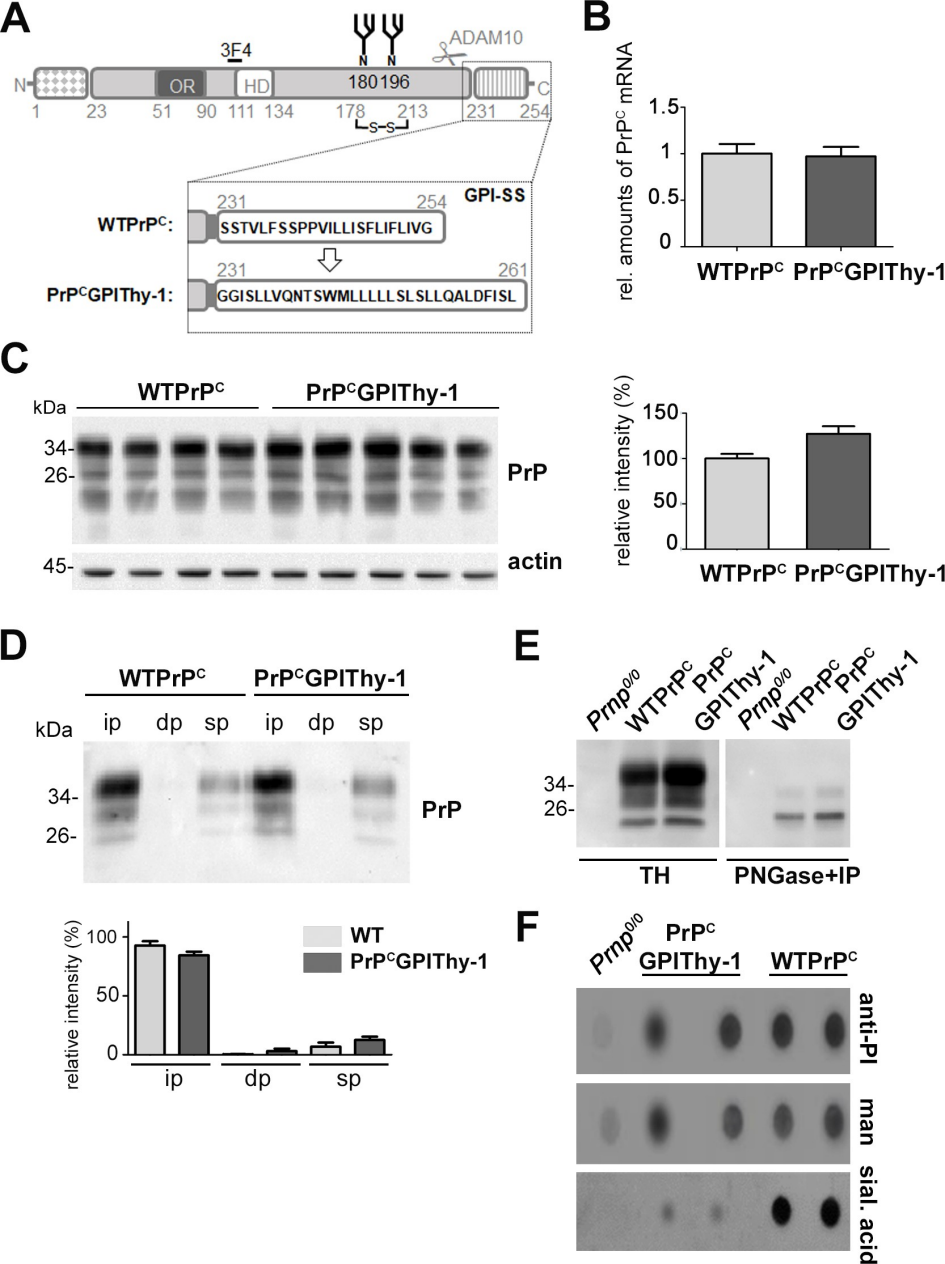
Biochemical characterization of PrP^C^GPIThy-1 mice. (A) Schematic representation of the PrP^C^GPIThy-1 fusion protein. PrP^C^ comprises an N-terminal and a C-terminal (GPI-anchor) signal sequence, both of them not being present in the mature protein. Positions of an octameric repeat region (OR), a hydrophobic domain (HD), two N-glycans (N), a disulfide bridge, the 3F4 tag and epitope (present in mouse lines L27 and L16) and of the cleavage site for the ADAM10-mediated shedding (scissors) are indicated. The substitution of the GPI-SS of PrP^C^ for the one of Thy-1 is shown in the dotted box. The substitution of the GPI-SS of PrP^C^ for the one of Thy-1 is indicated. (B) Relative amount of PrP^C^ mRNA from WTPrP^C^ (n = 5) and PrP^C^GPIThy-1 (n = 5) measured by RT-qPCR. WTPrP^C^ mRNA is set to one. Error bars are SEM. (C) Representative western blot showing PrP^C^GPIThy-1 protein expression in brain compared to WTPrP^C^. Bar chart shows the mean of PrP relative intensity related to actin intensity (used as a loading control). WTPrP^C^ is set to 100%. Error bars are SEM. (D) Triton X-114 phase partitioning assay. Both, WTPrP^C^ and PrP^C^GPIThy-1 were mainly found in the insoluble phase (ip), indicating that both carry a GPI-anchor as described [28] (dp: detergent phase; sp: soluble phase). Diagram shows the relative signal intensity of PrP from 3 independent experiments. WTPrP^C^ ip is set to 100%. Error bars are SEM. (E) Representative western blot showing total homogenates (TH) from PrP KO (*Prnp*^0/0^, used as a negative control), WTPrP^C^ and PrP^C^GPIThy-1 brains used for PNGase treatment (to eliminate N-glycans) followed by PrP immunoprecipitation (IP), shown in the adjacent western blot. PNGase+IP treated samples were then used to isolate the GPI-anchors, showed in (F). (F) Dot blot analysis of the GPI-anchors from deglycosylated, immunoprecipitated PrP and PK digested samples from mouse brain. Phosphatidylinositol (PI), mannose (man) and sialic acid (sial. acid) were detected as described in Methods. Note that the amounts of PI and mannose are similar between PrP^C^GPIThy-1 and WTPrP^C^ whereas sialic acid is almost absent in PrP^C^GPIThy-1. Sialic acid background signal in PrP^C^GPIThy-1 could be explained by deglycosylation not being 100% achieved as shown in blot in (E).

We generated four lines of transgenic mice (PrP^C^GPIThy-1) expressing PrP^C^ with the GPI-SS of Thy-1 (Fig 1A) which were backcrossed into PrP knockout (*Prnp*^0/0^ in C57/Bl6 background) mice. Two of the lines, L27 and L16, had the 3F4 tag [26]. However, since this modification can increase prion disease incubation time [27], we also generated two other lines, L150 and L159, without the 3F4 tag, of which L150 was used for prion inoculation experiments. A WTPrP^C^ line was generated with littermates of the founders, which did not contain the transgene but had an identical genetic background. WTPrP^C^ mice were backcrossed with C57/Bl6 mice to avoid differences in prion incubation times depending on the genetic background. All lines described here developed normally for the period observed (>350 days) and did not present any obvious phenotypic alterations.

**Fig 2:**
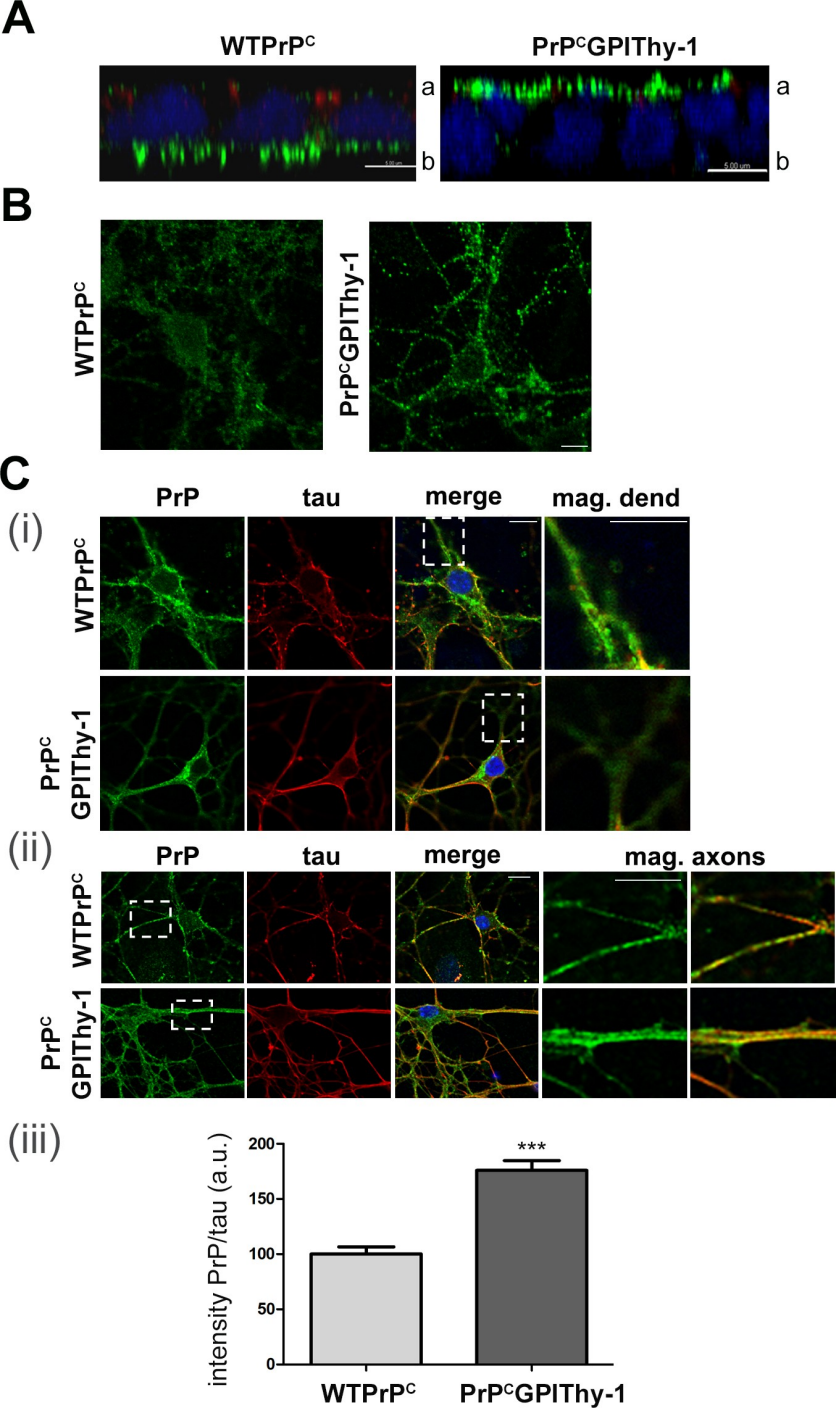
PrP^C^GPIThy-1 shows altered sorting. (A) Confocal microscopy showing expression of WTPrP^C^ and PrP^C^GPIThy-1 (green) in fully polarized MDCK epithelial cells. ZO1 (red) is expressed at the tight junction and delimitates the apical (a)/basolateral (b) side. Note that PrP^C^GPIThy-1 relocalizes to the apical side compared to WTPrP^C^, which is predominantly basolaterally located (scale bar is 5 μm). (B) Confocal microscopy of primary neuronal cultures stained with an antibody against PrP (POM1; green) under non-permeabilizing conditions. Both WTPrP^C^ and PrP^C^GPIThy-1 are expressed at the plasma membrane (scale bars are 10 μm). (C) Representative confocal microscopy pictures of primary neuronal cultures stained with antibodies against PrP (POM1; green) and tau (red). (i) The squares indicate selected areas of the dendrites (where tau is absent) showing decreased PrP^C^GPIThy-1 amounts whereas staining is present in WTPrP^C^. (ii) Same staining as in (i) but focusing on tau-positive axons (the squares indicate magnifications where the relative increase in PrP staining at the axons in PrP^C^GPIThy-1 neurons can be observed). (iii) Bar diagram of a semi-automated quantification showing that the amount of PrP^C^GPIThy-1 present in tau-positive axons is significantly increased compared to WTPrP^C^ (***p = 0.0001).

Analyses of PrP^C^ expression by RT-qPCR and western blot (Fig 1B and 1C) showed similar PrP^C^-levels for line L27 and a two-fold increase of PrP^C^ levels for line L16 (S1A Fig) when compared to wild-type mice (WTPrP^C^). Both lines of transgenic mice also showed a similar pattern of expression in different organs as WTPrP^C^ (S1B Fig). L27 was used for the detailed biochemical characterization of PrP^C^GPIThy1 in mice (Figs 1 and 2).

To assess whether a proper GPI-anchor was added, we performed a Triton X-114 phase separation assay [28]. We found that PrP^C^GPIThy-1, like WTPrP^C^, is mainly present in the insoluble pellet, indicating GPI-anchorage (Fig 1D). Thy-1 and PrP^C^ are both DRM residents, but share different lipid domains at the plasma membrane that can, in principle, be isolated by differential detergent solubilization [29]. We hypothesized that the exchange of the GPI-SS of PrP^C^ for the one of Thy-1 would alter the lipid subdomain localization of the protein. We isolated lipid rafts from frontal cortex using Brij 96 (0.5%) and sodium deoxycholate (NaDOC, 0.5%), as these detergents were described to discriminate between lipid subdomains [30]. As shown in S2A Fig, both WTPrP^C^ and PrP^C^GPIThy-1 were mainly solubilized and present at the bottom of the gradient (fractions 11 and 12), whereas flotillin, a marker of lipid rafts, and Thy-1 were found in the upper fractions of the gradient under these conditions (S2B Fig). We myelin-depleted the sample before detergent incubation, as myelin interferes with proper detergent solubilization. After solubilization with either a Brij 96 (0.5%)/NaDOC (0.5%) mixture or with Brij 98 (1%) as previously described [31], we obtained similar results, with the majority of WTPrP^C^ and PrP^C^GPIThy-1 solubilized and present at the bottom of the gradient (S2C Fig).

Since the PrP^C^ GPI-anchor contains sialic acid and the Thy-1 GPI-anchor does not, we assessed sialic acid content of both GPI-anchors. WTPrP^C^ and PrP^C^GPIThy-1 brain homogenates were first deglycosylated (as the N-glycans of PrP^C^ also contain sialic acid), immunoprecipitated with the PrP^C^-directed POM1 antibody (Fig 1E) and, after proteinase K (PK) digestion, their GPI-anchors were dot-blotted. As shown in Fig 1F, the amounts of phosphatidylinositol (PI) and mannose (man) as controls did not differ substantially between samples, whereas the amount of sialic acid was drastically reduced in PrP^C^GPIThy-1, showing a GPI-anchor composition similar to Thy-1 (S3 Fig). Thus, although the sublipid domain occupancy was not altered, the composition of the GPI-anchor was indeed changed. This suggests that the GPI-SS dictates the composition of the resulting GPI-anchor.

### Altered sorting of PrP^C^GPIThy-1 compared to WTPrP^C^

As we have described [11], PrP^C^GPIThy-1, in contrast to WTPrP^C^, is mainly sorted to the apical compartment when expressed in a polarized model of epithelial cells, thus behaving like Thy-1 (Fig 2A). In neurons, PrP^C^ is mainly present in the somatodendritic compartment whereas Thy-1 is more uniformly present in cell bodies and axons [29].

We could observe that, under non-permeabilizing conditions, PrP^C^GPIThy-1 was found at the plasma membrane similarly to WTPrP^C^, indicating that GPI-SS does not act on overall plasma membrane localization (Fig 2B). Next, we evaluated the presence of PrP^C^ in axons of primary hippocampal neurons isolated from PrP^C^GPIThy-1 and WTPrP^C^ mice (Fig 2C). PrP^C^GPIThy-1 shows a significantly higher degree of localization in tau-positive axons than WTPrP^C^ when assessed in primary neurons (100 ± 6.5% for WTPrP^C^ vs. 176 ± 8.8% for PrP^C^GPIThy-1; ***p = 0.0001; unpaired t-test).

### PrP^C^GPIThy-1 mice challenged with prions show delayed clinical presentation and different neuropathology when compared to WTPrP^C^ mice

**Fig 3:**
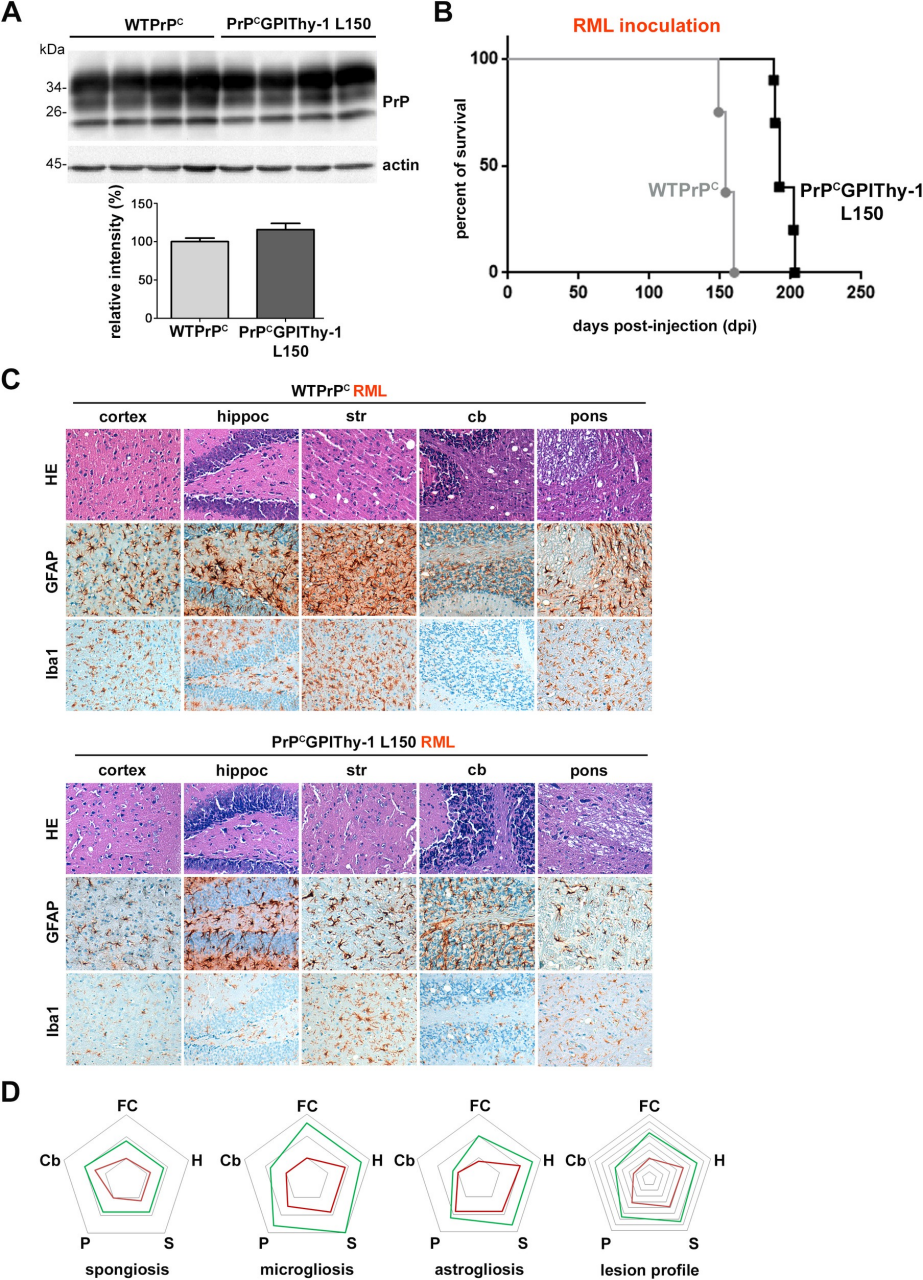
PrP^C^GPIThy-1 L150 mice show a delay to terminal disease and altered neuropathology upon prion infection. (A) Representative blot of PrPGPIThy-1 L150 (generated for infection experiments) and WTPrP^C^ mouse brain homogenates showing no relevant differences in the amount of PrP^C^GPIThy-1 and WTPrP^C^ in the brain. Chart underneath shows mean relative intensity of PrP referred to actin where WTPrP^C^ samples are set to 100% as a reference (error bars are SEM; n = 5 for each genotype). (B) Kaplan-Meier survival curve. Mice were inoculated intracerebrally (i.c.) with RML prions and sacrificed at terminal disease. Note the significant delay (****p<0.0001) between PrP^C^GPIThy-1 L150 (n = 10) and WTPrP^C^ mice (n = 8) incubation time. (C) Neuropathological characterization of terminally diseased mice. Note that PrP^C^GPIThy-1 L150 mouse brains show a general decrease in spongiosis as observed with HE staining. Gliosis is also decreased in the transgenic mice as manifested by reduced GFAP (astrocytes) and Iba1 staining (microglia) in all the studied regions. (D) Lesion profile after semiquantitative assessment (n = 3 or n = 4 for each genotype) of spongiosis and gliosis (FC: frontal cortex; H: hippocampus; S: striatum; P: pons; Cb: cerebellum). Note the general decrease in lesion severity for the PrP^C^GPIThy-1 L150 mice (red line), compared to WTPrP^C^ profile (green line).

As stated in material and methods, for prion inoculation experiments we generated new lines of transgenic mice lacking the 3F4 tag (S1 Table). As shown in Fig 3A and S4 Fig, we obtained two lines, PrP^C^GPIThy-1 L150 and PrP^C^GPIThy-1 L159, with different expression levels of the transgene. We chose L150 for prion infection as these mice expressed amounts of PrP^C^GPIThy-1 equal to endogenous PrP^C^ levels in WTPrP^C^ mice (Fig 3A and S4A Fig). As shown in Fig 3B, terminal prion disease in transgenic animals inoculated with mouse-adapted RML prions occurred at 195 ± 2 days post-infection (dpi; SEM; n = 10), thus showing a significant delay compared to WTPrP^C^ mice (155 ± 1.6 dpi (SEM; n = 8); Log Rank (Mantel-Cox) ****p<0.0001). The delay was independent of the prion strain as PrP^C^GPIThy-1 L150 mice inoculated with another strain (22L; S5 Fig), also presented with a significant delay in incubation time (156 ± 3 dpi; (n = 5) compared to 144 dpi in WTPrP^C^ (n = 5); Log Rank (Mantel-Cox) **p = 0.003). Interestingly, transgenic RML-infected mice, although showing a delay to terminal disease, presented a more rapid disease progression after clinical onset than the WT mice (duration of clinical phase: 20 ± 2 dpi in PrP^C^GPIThy-1 L150, compared to 40 ± 2 dpi in WT [32]).

Sagittal brain sections of terminally prion-diseased WTPrP^C^ and PrP^C^GPIThy-1 L150 mice were neuropathologically examined. As shown in Fig 3C, RML prion-infected PrP^C^GPIThy-1 L150 mice presented with decreased spongiosis and less severe astro- and microgliosis when compared to WTPrP^C^. By analyzing the lesion profile (as described elsewhere [33], Fig 3D), we found an overall decrease in the severity of prion-associated lesions, namely less spongiosis in all the studied areas, reduced microgliosis (less pronounced in the cerebellum) and astrogliosis (although with similar amounts in pons).

In another set of experiments, we also inoculated PrP^C^GPIThy-1 L16 mice with RML and 22L mouse-adapted prions. RML-inoculated transgenic mice reached terminal disease after 400 ± 56 dpi (n = 10), whereas WTPrP^C^ mice became terminally sick at 155 ± 1.6 dpi (n = 8), thus also showing a highly significant delay for PrP^C^GPIThy-1-expressing mice (Log Rank (Mantel-Cox)****p<0.0001; S6A Fig). Upon inoculation with 22L prions, PrP^C^GPIThy-1 L16 mice again presented with a significant delay (200 ± 23 dpi; n = 5) compared to controls (144 ± 1 dpi; n = 5; Log Rank (Mantel-Cox) **p<0.003; S6A Fig). Although this drastic delay to terminal disease in PrP^C^GPIThy-1 L16 mice may partially be explained by the presence of the 3F4 tag, decreased lesion severity in these mice was conspicuously similar to the infected PrP^C^GPIThy-1 L150 mice lacking the 3F4 tag, with an overall decrease in spongiosis and glial activation (S6B Fig).

### The PrP^Sc^ pattern differs between PrP^C^GPIThy-1 and WTPrP^C^ mice

**Fig 4:**
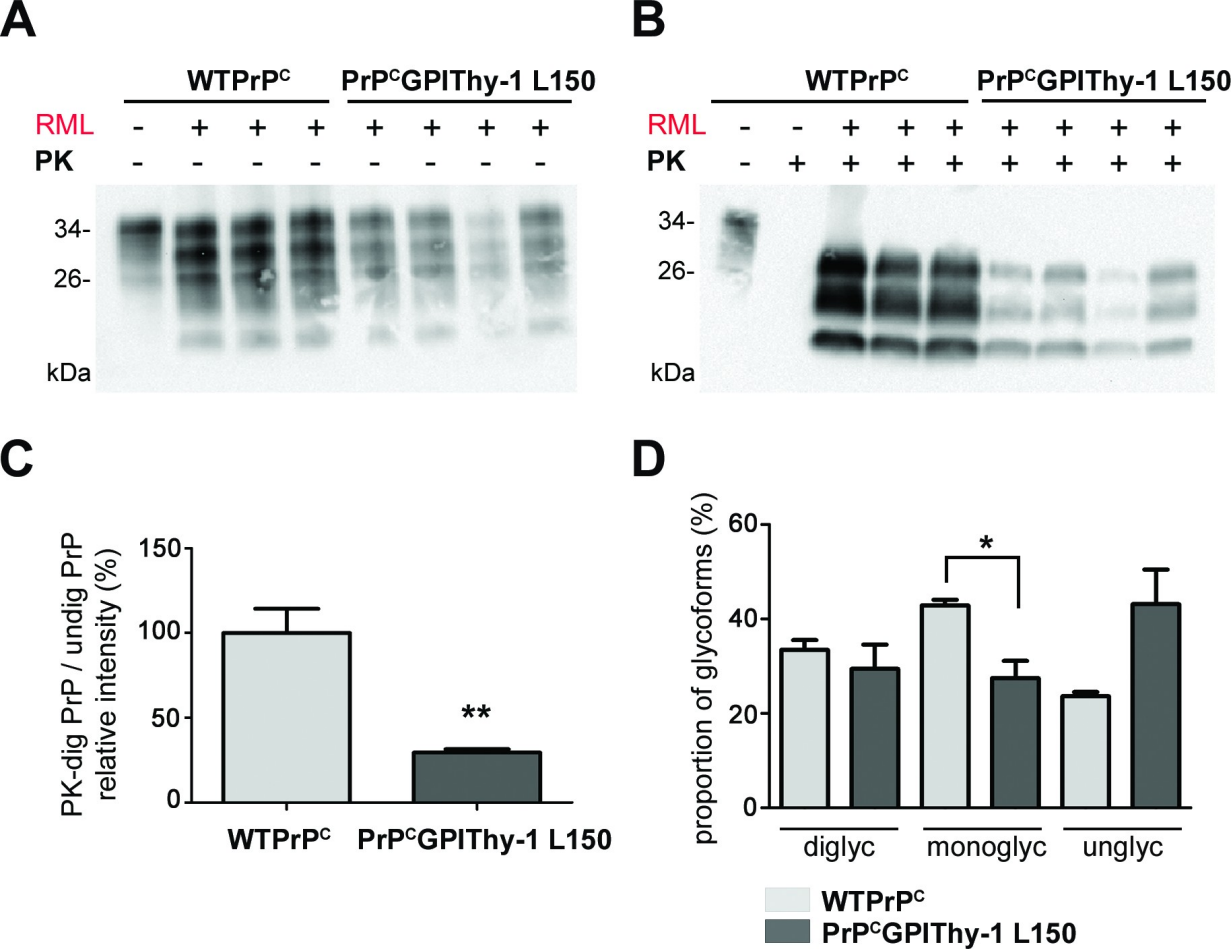
The amount of PK-resistant PrP is decreased, and the glycopattern is changed in PrP^C^GPIThy-1 L150 mouse brains after RML prion infection. Representative blots of total brain homogenates from terminally sick WTPrP^C^ and PrP^C^GPIThy-1 L150 mice infected with RML prions, without (A) and after (B) PK digestion at 37°C and detected with POM1 antibody. Note that there is a significant overall decrease in the total amounts of PrP^C^GPIThy-1 in the infected brains (n = 4) which is more conspicuous after digestion with PK (B). (C) Bar chart representing the mean relative intensity of PK-digested samples related to the undigested amounts. Note the significant decrease in the amount of resistant PrP^Sc^ for PrP^C^GPIThy-1 compared to WTPrP^C^ (**p = 0.0021). (D) Quantification of the glycoform banding pattern of PrP^Sc^. The intensity of each band in the PK-digested samples was measured and referred to the total amount of PrP as a percentage. The glycopattern is significantly changed in PrP^C^GPIThy-1 L150 after RML infection, where the monoglycosylated isoform is less PK-resistant compared to WTPrP^C^ (*p = 0.019).

To investigate the amount and type of PrP^Sc^, we performed biochemical analyses of brain samples from terminally prion-diseased mice. Interestingly, PrP^C^GPIThy-1 L150 mice infected with RML showed decreased amounts of total PrP (Fig 4A) with significantly less PrP^Sc^ compared to WTPrP^C^ mice (100 ± 14.26% for WTPrP^C^ mice vs. 29.4 ± 2% for PrP^C^GPIThy-1 L150 mice; Fig 4B and 4C, **p = 0.0021; unpaired t-test). Likewise, RML-infected PrP^C^GPIThy-1 L16 mice also had significantly less PK-resistant PrP^Sc^ (S6C Fig). The reduction in total PrP was not due to an age-dependent decrease in PrP^C^ expression as we did not observe significant differences between 20 and 40 weeks-old PrP^C^GPIThy-1 L150 mice (S7A Fig). Moreover, no significant differences in total PrP levels were observed between terminally prion-diseased (around 30 weeks old) and non-infected PrP^C^GPIThy-1 L150 mice (40 weeks old; S7B(ii) Fig). Taken together, these findings suggest that the relative decrease of total PrP after infection in PrP^C^GPIThy-1 is due to less efficient conversion to PrP^Sc^ in these mice compared to WTPrP^C^. In addition, the PrP^Sc^ glycopattern was also changed in prion-diseased PrP^C^GPIThy-1 L150 mice, which showed significantly less monoglycosylated PrP^Sc^ (Fig 4D, 42.9 ± 1.9% in WTPrP^C^ vs. 27.5 ± 3.6% in PrP^C^GPIThy-1 L150; *p = 0.0173, unpaired t-test).

### Proteolytic shedding of PrP^C^GPIThy-1 is reduced compared to WTPrP^C^

**Fig 5:**
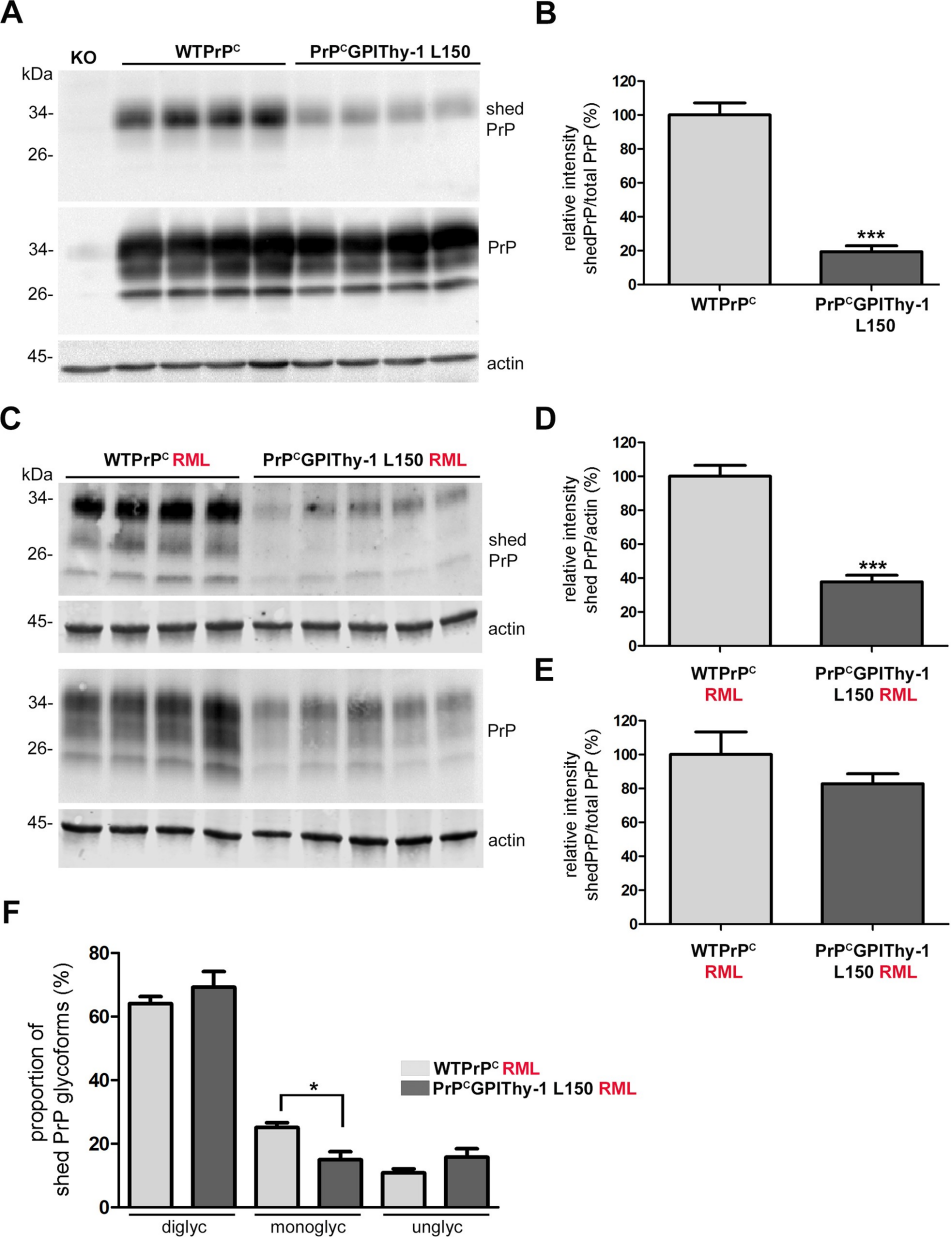
Shedding of PrP is decreased in PrP^C^GPIThy-1 L150 mice but increases after RML infection. (A) Representative western blot showing shed PrP (detected with our new antibody described in [36]) and total PrP levels (detected with POM1). The blots for shed PrP and total homogenates were run in parallel (n = 4 for each genotype). The diglycosylated band is preferentially shed in both WTPrP^C^ and PrP^C^GPIThy-1 L150 brains, but in the latter, shedding is reduced to 27% (***p = 0.0005) as shown in the bar chart (B). To quantify, each blot (shed and total PrP) was first referred to actin prior to the relative quantification of shed PrP referred to total PrP. WTPrP^C^ is set to 100% and error bars are SEM. (C) Representative western blot of shed PrP and total PrP from RML infected brain homogenates of terminally sick mice (n = 4 for WTPrP^C^ and n = 5 for PrP^C^GPIThy-1 L150). Note that in the infected brains all the isoforms are shed, in both WTPrP^C^ and PrP^C^GPIThy-1 L150. (D) Bar chart showing the quantification of shed PrP referred to actin. WTPrP^C^ is set to 100%. Note that shedding in PrP^C^GPIThy-1 L150 is still decreased compared to WTPrP^C^ (***p = 0.0004). (E) Bar chart showing that when shed PrP is referred to the total PrP in RML infected mice, there are no significant differences in the relative amount of shedding, implying a relatively increased shedding in infected PrP^C^GPIThy-1 L150 brains. Blots were run in parallel and first referred to its actin. WTPrP^C^ is set to 100%. (F) Quantification of the shed PrP glycopattern. Each band intensity was referred to the total amount of shed PrP. In infected brains, the monoglycosylated isoform is significantly less shed in PrP^C^GPIThy-1 L150 mice (*p = 0.0158).

PrP^C^ undergoes several physiological cleavages which are highly conserved through evolution, indicating essential functions [34]. We have previously shown that the metalloprotease ADAM10 is the responsible protease for the shedding of PrP^C^ *in vivo* [35] and that impaired shedding has significant consequences for prion diseases [32]. Thus, to investigate the possible mechanisms implicated in the clinical delay and the altered neuropathology, we decided to analyze the shedding of PrP^C^GPIThy-1. We have recently generated an antibody (sPrP^G228^) that specifically recognizes shed PrP (as the antibody is directed against the carboxy terminus Gly228 only exposed upon ADAM10-mediated cleavage) allowing for the direct detection of shed PrP in mouse brain homogenates [36]. By using this antibody, we found a significantly decreased shedding in PrP^C^GPIThy-1 L150 and PrP^C^GPIThy-1 L159 mice compared to WTPrP^C^ (Fig 5A and 5B; 100 ± 72% in WTPrP^C^ vs. 19.37 ± 3.5% in PrP^C^GPIThy-1 L150; ***p = 0.0005; S8A and S8B Fig, 100 ± 14.8% in WTPrP^C^ vs. 37.5 ± 3.9% in PrP^C^GPIThy-1 L159; *p = 0.015; unpaired t-test). This indicates that the altered GPI-anchor and/or localization of PrP^C^GPIThy-1 interferes with the ADAM10-mediated release from the plasma membrane. Remarkably, in terminally diseased RML-infected PrP^C^GPIThy-1 mice, even though total PrP shedding is decreased compared to WTPrP^C^ (Fig 5C and 5D, 100 ± 6.4% in WTPrP^C^ vs. 37.77 ± 3.9% in PrP^C^GPIThy-1 L150; ***p<0.0001; unpaired t-test), it does not show differences when it is referred to total PrP amounts (Fig 5E). This indicates a relative increase of PrP^C^GPIThy-1shedding upon infection, which was further confirmed by comparing levels of shed PrP^C^GPIThy-1 between non-infected and infected mice (S7B(i) Fig; 100 ± 11.8% in PrP^C^GPIThy-1 L150 vs. 272 ± 21% in PrP^C^GPIThy-1 L150 RML infected; ***p = 0.0004; unpaired t-test). We also observed that in brains of both, RML-infected WTPrP^C^ and PrP^C^GPIThy-1 L150 mice, all PrP glycoforms could be shed (Fig 5C), contrasting with the almost exclusive shedding of diglycosylated PrP^C^ in non-infected samples (Fig 5A and S7 Fig, also characterized in [36]). In PrP^C^GPIThy-1 L150 mice, the monoglycosylated isoform is significantly less shed compared to WTPrP^C^ (Fig 5F, 25.08 ± 1.6% in WTPrP^C^ vs. 15 ± 2.5% in PrP^C^GPIThy-1 L150; *p = 0.0158; unpaired t-test), possibly reflecting its decreased presence in these infected brains as shown in Fig 4D. In conclusion, ADAM10-mediated shedding is significantly altered for PrP^C^GPIThy-1 compared to WTPrP^C^ under both physiological and pathological conditions. In order to properly interpret these results, one should keep in mind that shed PrP (made visible by the highly sensitive sPrP^G228^ antibody) represents only a minor fraction of the total PrP pool (repeated own observations and [37]).

### Decreased ERK activation in brains of PrP^C^GPIThy-1 L150 mice after RML infection

**Fig 6:**
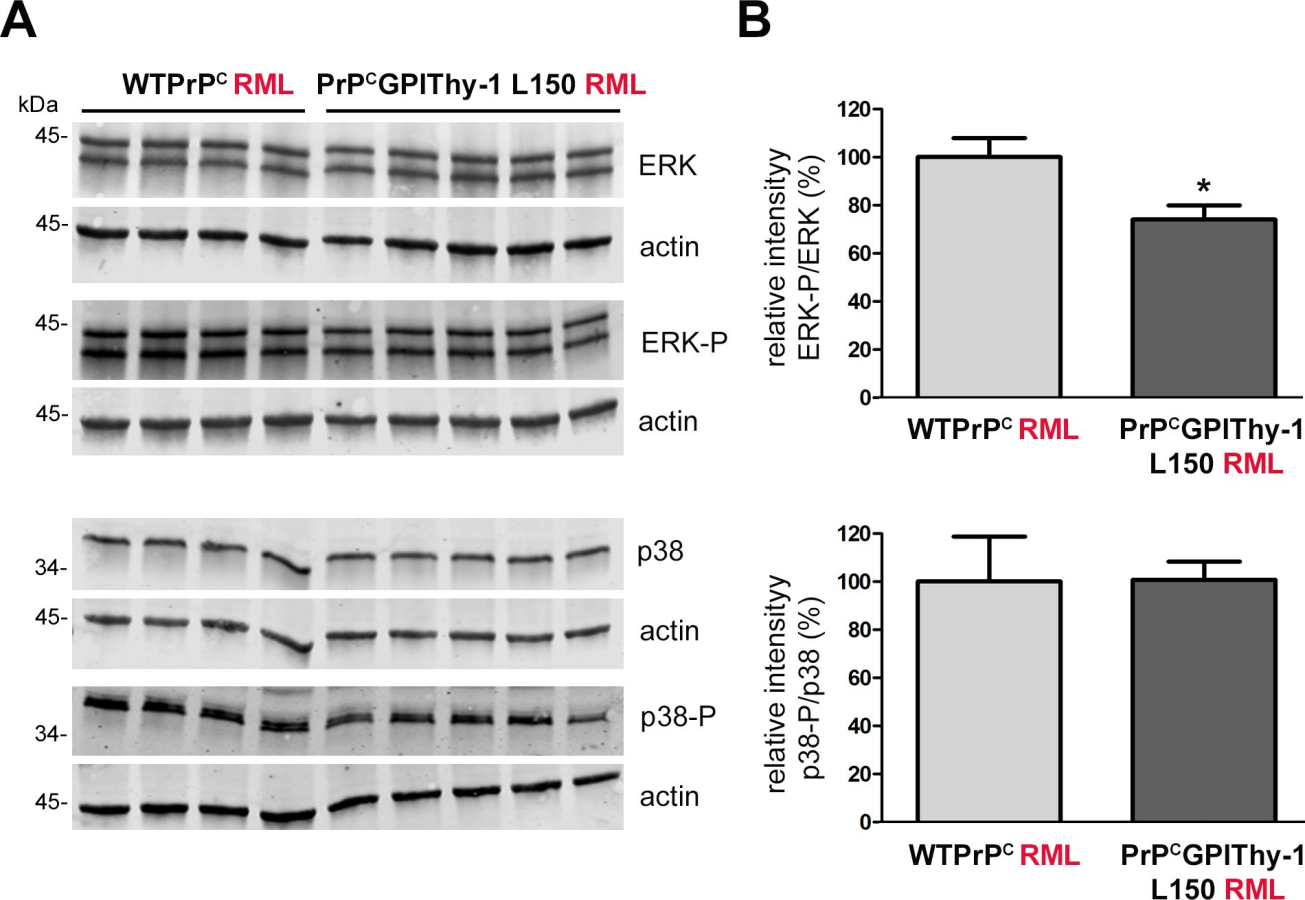
Phosphorylated ERK (ERK-P) is decreased in terminally prion-diseased PrP^C^GPIThy-1 L150 mice. **(**A) Representative western blots of total brain homogenates from prion diseased mice infected with RML prions, incubated with antibodies against total ERK and ERK-P (upper part) as well as total p38 and p38-P (lower part). (B) Quantifications of the relative intensity of ERK-P signal related to total ERK signal and p38-P signal (upper part) related to the total p38 signal (lower part). (WTPrP^C^, n = 4; PrP^C^GPIThy-1, n = 5). For the quantification, each signal was first related to the corresponding actin signal. Note that there is a significant decrease in ERK-P signal (*p = 0.031) in PrP^C^GPIThy-1 RML infected mice compared to WTPrP^C^ mice, whereas no changes are observed for p38-P.

Activation of ERK1/2, a member of the mitogen-activated protein kinase (MAPK) family, can promote the formation of PrP^Sc^ in a cell model of prion infection [38]. In the same study, it was demonstrated that activation of other MAPKs, such as p38 or JNK, has an adverse effect on the formation of PrP^Sc^. We have recently shown that a C-terminal deletion retains PrP in the secretory pathway, leading to p38 activation and neuronal death [39]. Since in the present study RML-infected PrP^C^GPIThy-1 L150 mice showed decreased PrP^Sc^ formation and increased survival, we performed western blot analyses to assess the status of ERK1/2 and p38 MAPK phosphorylation. As shown in Fig 6A and 6B, clinically terminal PrP^C^GPIThy-1 L150 mice presented a significant decrease of phosphorylated ERK1/2 (*p = 0.031) when compared to WTPrP^C^. Phosphorylation of p38 instead was not significantly changed at terminal disease.

### PrP^C^GPIThy-1, like WTPrP^C^, mediates toxic PrP^Sc^-associated signaling

**Fig 7:**
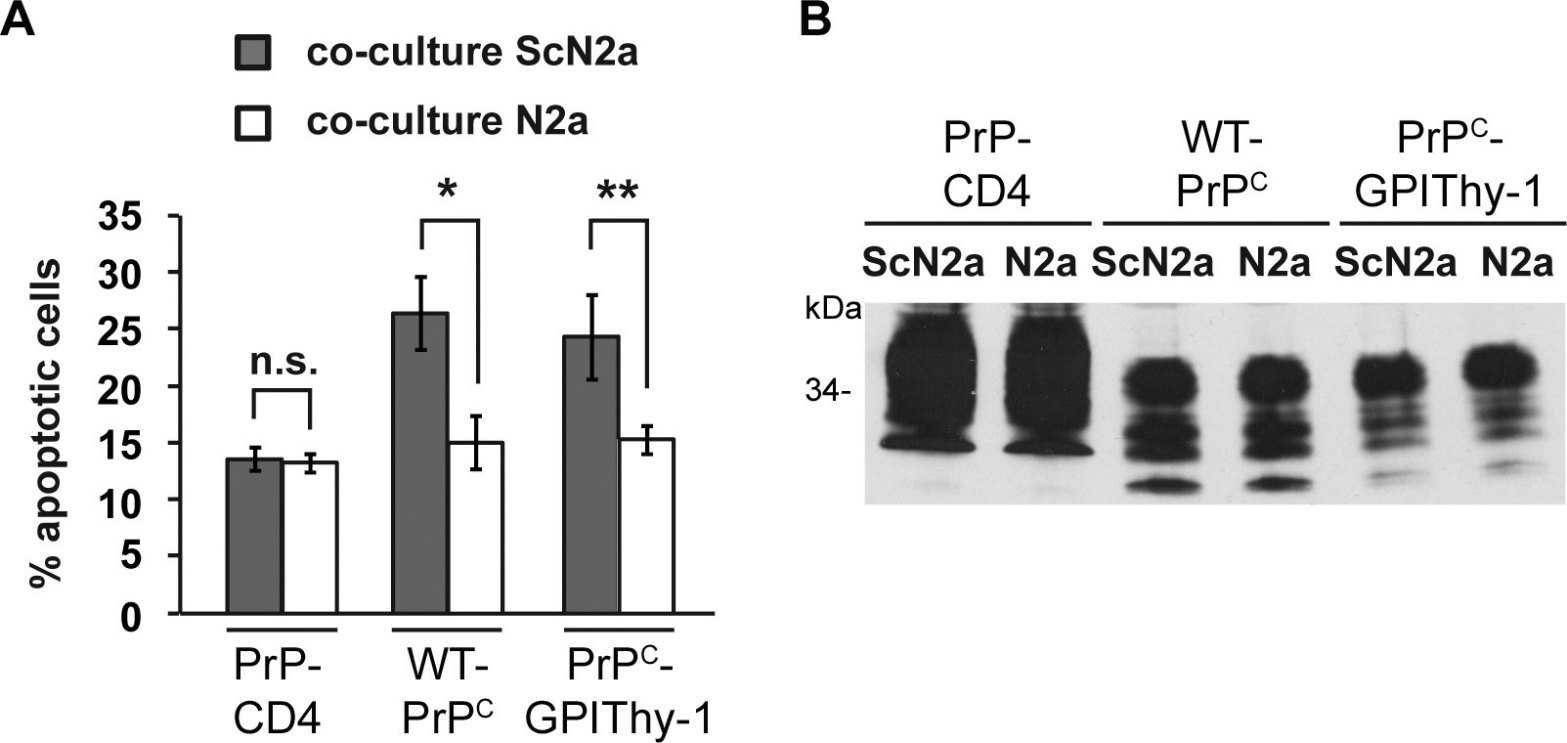
PrP^C^GPIThy-1 can induce prion-associated apoptosis in cells. (A) SH-SY5Y cells expressing the indicated PrP constructs (PrP-CD4 is a transmembrane version of PrP and served as a negative control) were co-cultured with ScN2a or N2a cells for 16 h. For quantification of apoptotic cell death, SH-SY5Y cells were fixed, permeabilized and stained for active caspase-3. Quantifications are based on triplicates of at least three independent experiments. (B) Comparable expression of PrP versions was confirmed by western blot using the anti-PrP antibody 3F4.

Since MAPK signaling was changed, and to assess whether PrP^C^GPIThy-1 is capable of mediating pro-apoptotic signaling induced by PrP^Sc^, we employed a cell culture model [40]. This assay is based on the co-cultivation of PrP^C^-expressing cells with chronically prion-infected cells that release PrP^Sc^ into the cell culture medium. As illustrated in Fig 7A, co-cultivation of SH-SY5Y cells expressing PrP^C^ with prion-infected mouse neuroblastoma (ScN2a) cells increased apoptotic cell death, as determined by activation of caspase-3. In contrast, SH-SY5Y cells expressing a PrP mutant containing a heterologous C-terminal transmembrane domain instead of the GPI-anchor (PrP-CD4) could be co-cultured with ScN2a cells without signs of apoptosis. Notably, PrP-CD4 also inhibits PrP^Sc^-formation in scrapie-infected neuroblastoma cells [18]. However, SH-SY5Y cells expressing PrP^C^GPIThy-1 transduce PrP^Sc^-mediated toxicity similar to WTPrP^C^. This supports that, despite an altered GPI-anchor composition and localization at the cell surface, PrP^C^GPIThy-1 is still able to contribute to the formation of signaling-competent complexes relevant to prion diseases.

## Discussion

**Fig 8:**
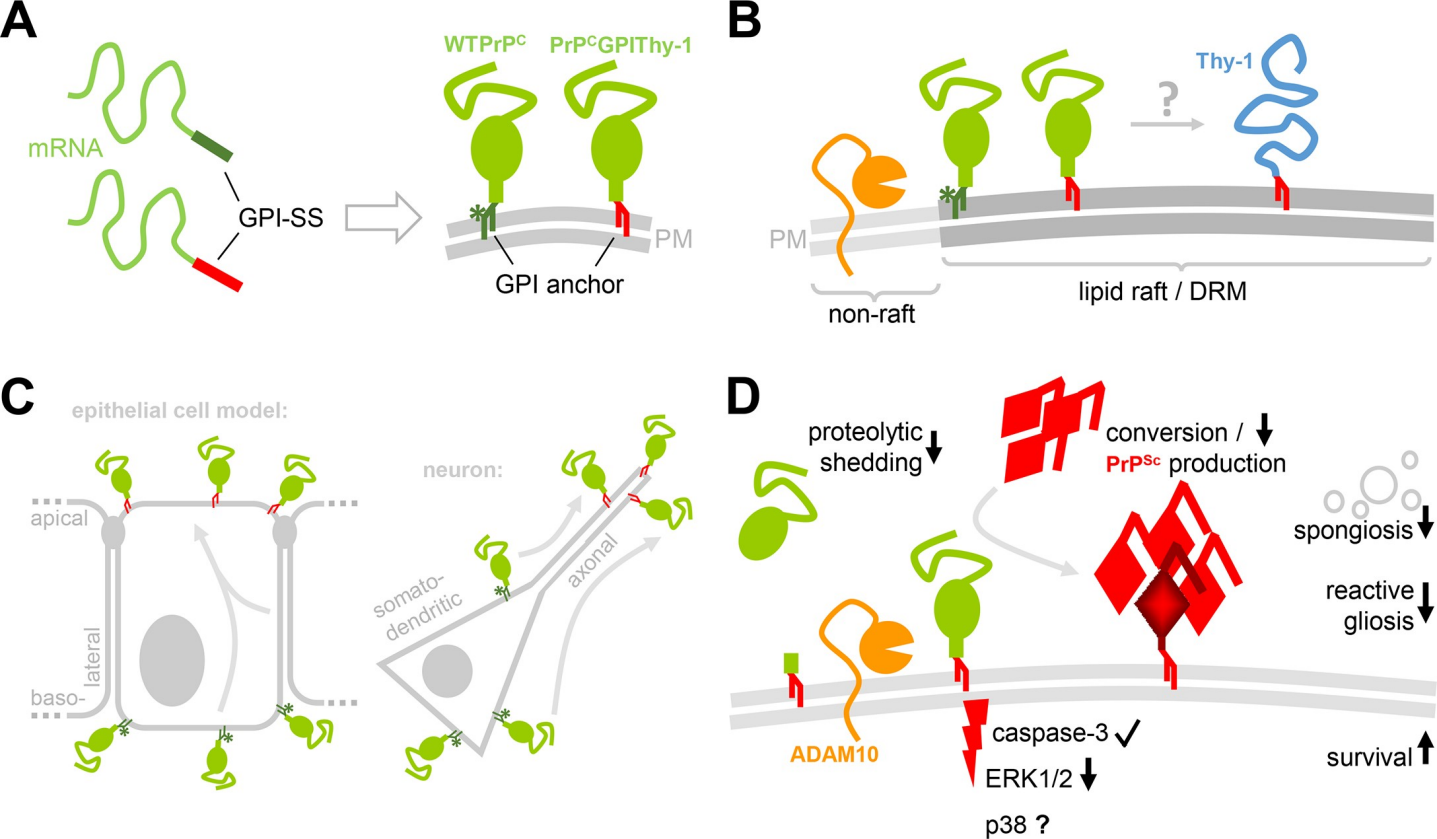
Graphical summary of research aim and key findings. (A) To study the role of the GPI-anchor signal sequence (GPI-SS) in determining localization and biology of a protein, the GPI-SS of PrP^C^ (dark green) was exchanged for the one of Thy-1 (red). Both proteins, WTPrP^C^ and the PrP^C^GPIThy-1 mutant, are expressed at the cell surface and comprise a GPI-anchor. A sialic acid modification (green asterisk) typical for the GPI-anchor of PrP^C^ is lacking in the GPI-anchor of PrP^C^GPIThy-1 (note its red GPI-anchor referring to the GPI-anchor of Thy-1 [in B]). This suggests that an altered GPI-SS in the mRNA results in a different GPI-anchor of the mature protein. (B) PrP^C^ (green) and Thy-1 (blue) are both known residents of lipid rafts/DRMs. While PrP^C^ is located in their periphery and able to leave and re-enter these membrane subdomains, Thy-1 has been shown to occupy more central regions therein. Despite several biochemical methods applied in our study, we were unable to demonstrate a relative re-distribution of PrP^C^GPIThy-1 towards Thy-1 compared to WTPrP^C^. (C) Nevertheless, the overall cellular sorting is altered with PrP^C^GPIThy-1 being relatively more transported towards the apical compartment compared to WTPrP^C^ with its predominant basolateral sorting in a polarized epithelial cell model such as MDCK cells. This also holds true in our transgenic mice and translates to an increased axonal sorting of PrP^C^GPIThy-1 in primary neurons compared to a mainly somatodendritic presence of PrP^C^ in wild-type neurons. (D) Altered GPI-anchor composition and sorting of PrP^C^GPIThy-1 results in different biological consequences (in comparison to WTPrP^C^): (i) Endogenous proteolytic shedding by the metalloprotease ADAM10 (orange) at the cell surface is reduced. (ii) Although PrP^C^GPIThy-1, in principle, is able to transduce PrP^Sc^-associated toxic signaling (e.g. via cleaved caspase-3), signaling via the MAP kinase ERK1/2 is reduced upon prion infection. Though not investigated here, p38 signaling may be reduced at early time points, contributing to delay to terminal disease (showed as p38?). Key hallmarks of prion-associated neuropathology are also altered in the transgenic mice including (iii) decreased PrP^Sc^ production and deposition, (iv) reduced vacuolization (spongiosis) of the brain parenchyma, and (v) reduced induction of astrocytes and microglia (reactive gliosis). These changes are accompanied with prolonged survival of the PrP^C^GPIThy-1 mice and support a relevant impact of the GPI-SS on a GPI-AP`s biology.

The GPI-anchor is a complex structure for the attachment of proteins to the outer leaflet of the plasma membrane that involves more than 20 proteins in its production [5]. It bestows the capacity to localize in lipid rafts (determining compartmentalization), and although it does not reach the intracellular space, it can confer the ability of signal transduction through transmembrane spanning partners [41, 42]. The signal sequence for GPI-anchor attachment has a wide range of sequence diversity and its participation in determining the final composition of the GPI-anchor itself is not known. It is assumed that remodeling of the core GPI-anchor depends on the protein it is attached to, and on the cell type by which it is synthesized [42]. In the present study, we have made several novel observations important not only for prion diseases but the biology of GPI-APs. We could show in a newly generated transgenic mouse model that (i) by changing the amino acids of the GPI-SS, the composition of the GPI-anchor is modified, resulting in a differential sorting and proteolytic processing of the protein. Upon infection with prions, these alterations (ii) are associated with significantly prolonged prion disease incubation time, and (iii) influence the conversion to PrP^Sc^ as well as the neuropathological presentation including decreased gliosis and spongiosis. Fittingly, we also found (iv) reduced activation of the ERK signal cascade during prion disease (please refer to Fig 8 for a graphical summary of the principal findings). To our knowledge, this study is the first to demonstrate the importance of the GPI-SS in determining a GPI-anchor composition and sorting of a GPI-AP in a mouse model. In the specific case of PrP^C^, this has a direct link to the pathophysiology of prion disease.

Our previous observation in a model of polarized epithelial cells already indicated that the GPI-SS plays a role in protein sorting. We observed that, by changing the GPI-SS of PrP^C^ for the one of Thy-1, PrP^C^GPIThy-1 was partially, yet significantly, relocalized from the basolateral to the apical side [11], confirming previous observations from others [12]. In the present work, we extended our observations to transgenic mice expressing PrP^C^GPIThy-1 on a Prnp knock-out background. PrP^C^GPIThy-1 is correctly expressed at the plasma membrane, showing identical N-glycosylation as WTPrPC, and is located in lipid rafts. In primary hippocampal neurons, we showed a 1.8-fold increase of PrP^C^GPIThy-1 in axons when compared to controls, despite equal PrP^C^ expression levels. Notably, we showed that by changing the GPI-SS, the biochemical composition of the GPI-anchor is altered, presenting with a loss of the sialic acid usually present in the GPI-anchor of PrP^C^. It has recently been shown that the sialic acid in the GPI-anchor of PrP^C^ has a role in the synaptic targeting of PrP^C^ in cultured cells [13]. Thus, when sialic acid from exogenously administered GPI-anchored PrP is depleted, or the lipid moiety is changed, PrP^C^ still partitions in DRMs but is no longer targeted to synapses. In vivo, we also observed that PrP^C^GPIThy-1 lacking sialic acid is differently sorted, but we observed an increase in axonal targeting. The fact, that not only the sialic acid is missing but also the GPI-SS was changed for PrP^C^GPIThy-1, may account for these differences.

The fact that in the GPI-anchor of PrP^C^GPIThy-1 not only the sialic acid is missing but also the SS-GPI anchor is changed, can account for these differences. Our mouse model provides the opportunity to address this on the molecular level.

In mammals, the sorting of GPI-APs is complex, and in epithelial cell models it appears to depend on (i) the presence of the GPI-anchor itself, (ii) its remodelling in the trans-Golgi network (TGN), (iii) its partitioning in DRMs, and (iv) the capacity to oligomerize in the Golgi, which in turn depends on the clustering of DRMs among others [7, 43]. In neurons, the signals implicated in the targeting of proteins either exclusively to dendrites/axons or to both sides, are not clear. For GPI-APs, such as Thy-1, it has been suggested that early interaction with DRM components (especially with sphingomyelin and cholesterol) is necessary for axonal delivery of Thy-1 in mature neurons [44]. According to our data and recent findings of others, it could be hypothesized that the GPI-SS itself directs the protein to different lipid environments, which then influence how the GPI-anchor will be remodeled in the TGN, thereby affecting its oligomerization capacity and, hence, its further sorting [7, 45]. Actually, PrP^C^ and Thy-1 are both located in DRMs but occupy different subdomains therein [30]. However, even after using different detergents previously reported to allow for discrimination between PrP^C^- and Thy-1-enriched DRM subdomains [29, 31], we were unable to observe differences in the distribution between PrP^C^GPIThy-1 and WTPrP^C^.

Nonetheless, support for a relocalization of PrP^C^GPIThy-1 to a different subdomain comes from our finding of reduced ADAM10-mediated shedding. This proteolytic cleavage at the cell surface is likely to occur at the interface between raft and non-raft regions, where–supported by the ability of PrP^C^ to leave and re-enter DRMs [46]–protease and substrate are thought to interact [34]. In that regard, the *in vivo* data presented here complement our recent finding of significantly decreased shedding of PrP^C^GPIThy-1 in N2a cells [36]. Relocalization of the prion protein from the periphery of rafts towards more central regions (where Thy-1 resides [29]) could, therefore, explain the reduced shedding in PrP^C^GPIThy-1 mice. It is interesting that, despite reduced shedding compared to WTPrP^C^ under normal conditions, upon prion infection this cleavage is relatively increased in PrP^C^GPIThy-1 mice. We and others have shown that shedding interferes with PrP^Sc^ formation and beneficially influences prion disease incubation times [32, 47, 48]. Thus, upregulation of shedding during prion disease could represent a protective feedback mechanism to lower PrP^Sc^ production and prolong survival in our transgenic mice.

Our further aim was to elucidate how the differential localization of PrP^C^GPIThy-1 impacts on prion disease. We intracerebrally inoculated our transgenic and WTPrP^C^ mice with prions. Upon RML-infection, PrP^C^GPIThy-1 mice showed (i) delayed onset of terminal disease, (ii) different neuropathological presentation with decreased spongiosis, gliosis, reduced PrP^Sc^ amounts, and an altered PrP^Sc^-glycotype pattern, (iii) relatively increased shedding, and (iv) a decrease in ERK phosphorylation. Although, as demonstrated by others [27, 49, 50] and now confirmed by us, the 3F4 tag prolongs prion disease incubation times, we included these mice (PrP^C^GPIThy-1 L16) as they showed several neuropathological characteristics that were likewise present in the non-3F4-tagged transgenic mice (PrP^C^GPIThy-1 L150), thus strengthening our overall results.

It is known that not only the presence of PrP^C^ but also its type of membrane anchorage is fundamental for the pathophysiology of prion disease. Briefly, (i) presence of extracellular PrP^Sc^ does not lead to prion-induced neurodegeneration in the absence of PrP^C^ on neurons [51, 52], (ii) cells expressing either anchorless PrP or PrP with a transmembrane domain instead of the GPI-anchor are resistant to prion infection [17, 18, 53], and (iii) prion-infected transgenic mice expressing low amounts of anchorless PrP do not show clinical symptoms of prion disease despite high titers of infectivity and high levels of PrP^Sc^ [21, 54]. When the expression of anchorless PrP is increased, this leads to delayed onset of disease and generation of a new prion strain [22, 55]. Remarkably, the type of GPI-anchor also affects PrP^Sc^ formation. Thus, when amino acids in the C-terminus or within the GPI-SS of the murine sequence are replaced by their cunicular homologs (with rabbits being naturally resistant to prion infection), this modified PrP^C^ cannot be converted to PrP^Sc^ in a cell culture model [56].

Moreover, the presence of sialic acid in the GPI-anchor can influence conversion as PrP with a desialylated GPI-anchor cannot be converted to PrP^Sc^ and can even stop an ongoing infection *in vitro* [57]. Thus, a desialylated GPI-anchor may modify the lipid environment leading to inhibition of the signal transduction associated with PrP^Sc^ neurotoxicity. Fitting to this, our model with a PrP mutant lacking sialic acid in its GPI-anchor also shows delay to terminal disease as well as altered signal transduction when compared to WTPrP^C^, highlighting the importance of the sialic acid modification of the GPI-anchor in prion disease. However, *in vivo* we found conversion of PrP^C^GPIThy-1 to be reduced, not completely abolished.

As stated above, the sialylation status of the PrP^C^ GPI-anchor modifies the lipid environment *in vitro* [58]. PrP^C^ and Thy-1 share different lipid environments, and it has been shown that the lipid environment composition is fundamental for conversion of PrP^C^ to PrP^Sc^ [59, 60]. PrP^C^-enriched lipid rafts isolated from rat brain have a significant increase in cholesterol compared to Thy-1-containing lipid rafts [30]. *In vitro*, desialylated PrP^C^ is found in lipid rafts with increased cholesterol content, probably stabilizing PrP^C^ in lipid rafts and increasing its half-life at the plasma membrane [58]. Although, by performing differential detergent extraction, we could not detect a differential distribution of PrP^C^/PrP^C^GPIThy-1 in lipid rafts, we cannot rule out that the lipid environment of PrP^C^GPIThy-1 is altered in a way that impairs the conversion to PrP^Sc^.

In prion-infected mice both, sialo and asialo GPI-anchored PrP^C^ forms, can be converted to PrP^Sc^ and are present in infected brains and spleens [61, 62]. Our results, where delay to clinical disease is associated with a significant decrease in the amount of PK-resistant PrP^Sc^, suggest that asialo forms can be converted *in vivo* but with comparably low efficiency. Interestingly, Katorcha *et al*. [63] have found that a decrease in PrP N-glycan sialylation leads to a different glycoform pattern after prion infection. They also observed that when prion-infected desialylated brain homogenates where inoculated to Syrian hamsters, there was a drop in infectivity and PK-resistance. Because in their experiments they used a sialidase that can also eliminate the sialic acid of the GPI-anchor [25], and in view of our present results, it would be interesting to study if the sialic acid of the PrP GPI-anchor also partially contributes to the observations of Katorcha *et al*.

Several other aspects may also contribute to reduced PrP^Sc^ conversion and prolonged survival of our PrP^C^GPIThy-1 mice. On the one hand, Nemoto *et al*. [64] have recently described that the behavior of Thy-1 and PrP^C^ at the plasma membrane is different, with PrP^C^ showing a slower membrane diffusion compared to Thy-1. This may increase the probability of homophilic interactions for WTPrP^C^ but not for PrP^C^GPIThy-1. Since the conversion of PrP^C^ to PrP^Sc^ occurs at the plasma membrane [65], this effect could favor interactions between WTPrP^C^ and critical PrP^Sc^ seeds, thus enhancing the propagation of PrP^Sc^. In contrast, the lateral diffusion behavior of PrP^C^GPIThy-1 may be more similar to that of Thy-1, thus decreasing the conversion rate. On the other hand, the GPI-anchor itself influences the structure of a given protein [41]. In the case of PrP^C^, this could result in structural hindrance and, thus, less efficient PrP^C^ to PrP^Sc^ conversion.

PrP^C^GPIThy-1 mice presented with significantly increased incubation times with two different prion strains, RML and 22L (with longest delay to terminal disease with RML prions). Several factors may account for that. On the one hand, each prion strain has different cellular tropism [66] and 22L is mainly associated to astrocytes where it mediates indirect neuronal damage. On the other hand, the fact that RML and 22L prions use different endocytic pathways to infect the cell [67], could indicate that PrP^C^GPIThy-1 follows an altered endocytic pathway that facilitates 22L over RML infection.

Intriguingly, after prion infection, we observed a decrease in the glial response in all PrP^C^GPIThy-1 lines compared to controls. Prion diseases, like other neurodegenerative diseases, are accompanied by an increase in microglia with a phagocytic phenotype and by reactive astrocytes. Microglia, the resident immune cells of the central nervous system, may act beneficial or detrimental in prion diseases [68, 69]. In the present study, a decreased amount of microglia in PrP^C^GPIThy-1 mice coincides with a delay to the terminal stage of prion disease, but further experiments would be needed to establish a direct correlation. Of note, a recent study has functionally linked ADAM10-mediated shedding of PrP^C^ to inflammatory responses and monocyte recruitment to the brain [70]. So, it is conceivable that reduced levels of shed PrP (as a potential chemoattractant and activating factor) account for the impaired glial response in our mice. With regard to astrogliosis, it has been shown that astrocytes (i) accumulate PrP^Sc^ early in the disease [71], (ii) can rapidly internalize, traffic, and spread PrP^Sc^ in cell culture [72, 73], and (iii) that astrocytic PrP^C^ supports the development of prion disease in mice [74]. Since astrogliosis is decreased in our transgenic mice, this could contribute to both, delay to terminal disease and reduced PrP^Sc^ deposition. Moreover, because destruction of the extracellular matrix due to factors released by microglia and astrocytes may lead to the vacuolization observed in prion diseases [75, 76], decreased gliosis in PrP^C^GPIThy-1 mice could also explain their low degree of spongiosis. Interestingly, lack of vacuolization of the gray matter was also observed in prion-infected mice expressing GPI-anchorless PrP [22].

The GPI-anchor is necessary for toxic signaling associated to PrP [40] and it seems plausible that an altered GPI-anchor and localization of PrP^C^ affect its association with signaling-competent, membrane-spanning binding partners and, thus, signaling outcome [77]. We observed that, *in vitro*, PrP^C^GPIThy-1 is as able as WTPrP^C^ to transduce neurotoxic signals, and, *in vivo*, we showed decreased ERK1/2 phosphorylation whereas p38 MAPK was unchanged at a terminal stage of disease. Using an *in vitro* model of prion infection, Fang *et al*. [78] recently showed that phosphorylation of p38 is an early event in synaptotoxicity and that PrP^Sc^ specifically targets post-synaptic PrP^C^. In our transgenic mice, PrP^C^GPIThy-1 is partially depleted from the dendritic compartment, and we observed a delay in the clinical onset. We did not detected a decrease in p38 phosphorylation but we cannot rule out the possibility that, at an earlier disease state, p38 activation in PrP^C^GPIThy-1 mice might be lower than in controls, thus contributing to delayed disease onset.

An increase in ERK1/2 signaling has been consistently observed in cellular [79–82], mouse [83] and hamster models of prion infection [84], and is probably related to neurodegeneration and cell death. Activated ERK1/2 also participates in the conversion of PrP^C^ to PrP^Sc^ *in vitro* [38]. Accordingly, it could be hypothesized that the decrease in ERK phosphorylation in our model delays clinical disease and reduces PrP^Sc^ deposition. How exactly PrP^C^GPIThy-1 affects MAPK signaling deserves further studies, but it is interesting that interfering with this signaling cascade is considered a potential therapeutic option [80, 81]. However, since astrocytes represent the main cell population that increases ERK phosphorylation after prion infection [83], it is also possible that the observed reduction is due to reduced astrogliosis in our transgenic mice.

Although PrP^C^GPIThy-1 mice present with extended survival and delay to clinical disease, we observed a faster progression to terminal disease compared to controls. Several factors can account for this. On the one hand, the different localization of PrP^C^GPIThy-1 may induce alternative signaling pathways not investigated in this study and these could follow a kinetic leading to rapid disease development once a critical threshold is reached [85]. On the other hand, glia is less activated (maybe due to decreased levels of PrP^Sc^), which may prolong survival (given less inflammatory brain injury) but could also contribute to toxicity as non-functional cells and PrP^Sc^ may not be efficiently eliminated. The final sum up of positive and negative factors may influence the complex findings observed in PrP^C^GPIThy-1 mice.

In conclusion, we showed that the GPI-SS influences GPI-anchor composition and localization of a GPI-AP *in vivo*. This study attributes a novel function to the GPI-SS in subcellular trafficking *in vivo* and sheds light on several molecular events underlying prion-associated neurodegeneration.

## Materials and methods

### Ethics statement

Animal experiments were approved by the *Behörde für Gesundheit und Verbraucherschutz* of the *Freie und Hansestadt Hamburg* (permit numbers 80/08, 38/07 and 84/13). All the procedures were performed under the guidelines of the animal facility of the *University Medical Center Hamburg-Eppendorf* and in compliance with the *Guide for the Care and Use of Laboratory Animals*. Mice used for prion infection were anesthetized with a mixture of Xylazin hydrochloride and Ketamine hydrochloride in 0.9% NaCl prior to intracerebral prion inoculation. To sacrifice the mice, first they were anesthetized with halothane followed by neck dislocation.

### Generation of transgenic PrP^C^GPIThy-1 mice

The generation of the PrP^C^GPIThy-1 construct was already described elsewhere [11]. To insert PrP^C^GPIThy-1 in the half-genomic expression vector (mPrPHGC, a generous gift from M. Groschup, Institute for Novel and Emerging Infectious Diseases at the Friedrich-Loeffler-Institut, Greifswald-Insel Riems, Germany)[86], a PmlI restriction site was inserted after the stop codon of PrP^C^GPIThy-1 DNA by using the following primers: F:5´-CCCAAGGAGAAA**CACGTG**CCCTCGAGGTCCTTC-3´; R: 5´-GAAGGACCTCGAGGG**CACGTG**TTTCTCCTTGGG-3´ (PmlI restriction site is in bold), with the QuickChange Lightning mutagenesis kit (Stratagene). PrP^C^ was excised from mPrPHG by AgeI and PmlI (Fast Digest, Fermentas). PrP^C^GPIThy-1 was cut with AgeI and PmlI and ligated into the mPrPHG. For pronuclear injection, the mPrPHG vector was cut with SalI and NotI and separated in an agarose gel. The pronuclear injection was performed at the Transgenic Mouse Facility (ZMNH, Hamburg). Positive animals for the transgene were selected by PCR with the following primers: F: 5´-ATGTGGACTGATGTCGGCCT-3´; R: 5’-CTTGGAGGAGGGAGAGGGAA-3’. Lines L27 and L16 were established, and animals were backcrossed to PrP^0/0^ mice (on a C57/Bl6 background). To generate the control line, a littermate not presenting the transgene was backcrossed with C57/Bl6 mice with the same backcrossing scheme as the transgenic mice.

To eliminate the 3F4 tag we used the PrP^C^GPIThy-1 in mPrPHGC as a template and we change the two methionine residues at position 108 and 111 for leucine and valine respectively by using QuickChange Lightning (Stratagene) and the following primers: F: 5´-CAAACCAAAAACCAAC**CTC**AAGCAT**GTG**GCAGGGGCTGCGGCAGC-3´; R: 5´-GCTGCCGCAGCCCCTGC**CAC**ATGCTT**GAG**GTTGGTTTTTGGTTTG-3´ (mutations are in bold).

### Quantitative real time PCR

RNA was extracted from mouse brain tissue using Precellys Lysing Kit (Bertin Technologies) and precooled QIAzol (Qiagen). Tissue (n = 7 and n = 5 for WTPrP^C^; n = 6 for PrP^C^GPIThy-1 L16; n = 5 for PrP^C^GPIThy-1 L27; n = 5 for PrP^C^GPIThy-1 L150) was homogenized for 30 s at 2,000 rpm in a dismembrator and subsequently centrifuged at 2,000xg for 2 min at room temperature. The supernatant was mixed with 200 μl chloroform and incubated at room temperature for 3 min following centrifugation for 15 min at 12,000xg. Total RNA was purified from the upper phase using RNeasy Mini Kit (Qiagen) according to the manufacturer’s instructions. RNA concentration and purity were determined using the NanoDrop system (Thermo Fisher Scientific). First strand cDNA was synthesized using 1 μg of total DNase-treated RNA using RevertAid H Minus First Strand cDNA Synthesis kit (Thermo Fisher Scientific). Real-time PCR reactions were performed in a volume of 10 μl, consisting of 10 ng cDNA, 2XSYBR GreenPCRMasterMix (Applied Biosystems), and 0.2 μM of each primer in Rotor Gene Q (Qiagen). For the detection of murine Prnp gene, the following primer pairs were used: 5´GGCCAAGGAGGGGGTACCCATAAT 3´ and 5´TAGTAGCGGTCCTCCCAGTCGTTGC 3´. The RPL gene (F: 5´CGGAATGGCATGATACTGAAGCC 3´; R: 5´TTGGTGTGGTATCTCACTGTAGG 3‘) was used as a reference to calculate relative expression levels of *Prnp* using ΔCT values.

### Inoculation of mice with RML and 22L mouse-adapted prions

Briefly, 8–10 weeks old WTPrP^C^ (n = 8), PrP^C^GPIThy-1 L16 (n = 10) and PrP^C^GPIThy-1 L150 mice (n = 10) mice were anasthesized and intracerebrally inoculated with 30 μl (corresponding to 3x10^5^ log LD_50_) of RML 5.0 prion inoculum. Moreover, WTPrP^C^ (n = 5), PrP^C^GPIThy-1 L16 (n = 5) and PrP^C^GPIThy-1 L150 (n = 5) mice were inoculated with 30 μl of 10% 22L prion inoculum. Mice were checked two to three times per week for clinical signs of prion disease and daily once clinical symptoms appeared.

### Western blot analysis

Frontal cortex samples were homogenized as a 10% in RIPA buffer (50 mM Tris-HCl pH8, 150 mM NaCl, 1% NP40, 0.5% Na-Deoxycholate, 0.1% SDS) containing a cocktail of protease and phosphatase inhibitors (Roche), left for 10 min on ice, and centrifuged for 5 min at 12,000xg. The protein content of the supernatant was assessed by colorimetric analysis (QuickStart Bradford 1x Dye, Biorad) following the instructions of the supplier. Samples were standardized to 1 μg/μl in 4x loading buffer (250mM Tris-HCl, 8% SDS, 40% glycerol, 20% β-mercaptoethanol, 0.008% Bromophenol Blue, pH 6.8), boiled for 5 min at 95°C and subjected to electrophoresis (20 μg per sample). Proteins were then transferred to nitrocellulose membranes (Biorad) and incubated with mouse monoclonal antibodies against PrP^C^ (POM1, 1:2.500; A. Aguzzi, Zurich, Switzerland) and actin (1:2,000; Millipore), ERK (1:1,000), ERK-P (1:1,000) p38 (1:1,000), p38-P (1:1,000) all from Cell Signaling Technologies; and rabbit polyclonal specific for shed PrP (1:1,000 [36]). Membranes were incubated with appropriate secondary antibodies and then developed either with Pierce ECL Western Blotting Substrate or Pierce Femto (Thermo Scientific) in a CD camera imaging system (BioRad) or with Odyssey Image system (Licor) and quantified with Image Studio software (Licor).

### Immunohistochemistry

Processing of samples, including cutting and staining of paraffin sections with HE, was performed as published [39]. For immunohistochemistry, all sections were stained using the Ventana Benchmark XT machine (Ventana, Tuscon, Arizona, USA). Deparaffinised sections were boiled for 30–60 min in CC1 solution (Ventana, Tuscon, Arizona, USA) for antigen retrieval. Primary antibodies were diluted in 5% goat serum (Dianova), 45% Tris-buffered saline pH 7.6 (TBS) and 0.1% Triton X-100 in antibody diluent solution (Zytomed, Berlin, Germany). Sections were then incubated with primary antibody POM1 (1:100), Iba1 (Dako, 1:2.000) or GFAP (Dako, 1:400) for one hour. Anti-mouse or anti-rabbit histofine Simple Stain MAX PO Universal immunoperoxidase polymer (Nichirei Biosciences) was used as secondary antibody. Detection of secondary antibodies was performed with an ultraview universal DAB detection kit from Ventana with appropriate counterstaining and sections were cover-slipped using TissueTek glove mounting media (Sakura Finetek).

The neuropathological assessment (n = 3 or n = 4 depending on the genotype) was conducted by three independent investigators in a blinded fashion by grading the samples 1 (mild) to 3 (severe) depending on the staining intensity or spongiosis.

### Triton X-114 assay

The assay was performed as previously described by Hooper *et al*. [28] which is a modification of the method described earlier by Pryde and Philips [87]. All the buffers contained a protease inhibitor cocktail (Roche). Briefly, frontal cortex samples (n = 3 for each genotype) were homogenized in 10 vol. of 0.32M sucrose in 50mM HEPES/NaOH pH 7.4, centrifuged for 15 min at 8,000xg and the supernatant further centrifuged at 26,000xg for 2h. The resulting pellet was resuspended in H buffer (10mM HEPES/NaOH pH 7.4) with the addition of 2% of pre-condensed Triton X-114 (Sigma-Aldrich) in a total volume of 200 μl (final concentration of 2 μg/μl). Samples were vortex mixed for 1–2 s, let on ice for 5 min and centrifuged again at 8,880xg at 4°C in a fixed angle rotor. The resulting pellet was washed with 0.2 ml H buffer, centrifuged at 8,800xg for 10 min at 4°C and the resulting pellet was resuspended in 180 μl of H buffer. This was kept as the Insoluble Pellet. The supernatant was layered over a 0.3 ml of 6% sucrose cushion in T buffer (10mM Tris-HCl pH 7.4, 0.15M NaCl) and 0.06% precondensed Triton X-114, incubated at 30°C for 3 min and further centrifuged at 3,000xg for 3 min in a swinging bucket rotor. The sucrose cushion was removed from the pellet (Detergent Phase), and the latter was resuspended in 180 μl of H buffer.

The supernatant (upper aqueous phase) was mixed with 0.5%(v/v) pre-condensed Triton X-114, vortexed 1–2 s, kept 5 min on ice, and further layered over a 0.3 ml 6% sucrose cushion in T buffer, incubated at 30°C for 3 min and centrifuged again at 3,000xg for 3 min in a swinging bucket rotor. The pellet was discarded, and the upper phase mixed again with 2% (v/v) pre-condensed Triton X-114 and processed again as described in the step before but without the sucrose cushion. After the last centrifugation, the supernatant was kept as final aqueous phase.

An equal amount of sample was then mixed with 4x loading buffer, and 30 μl of sample was subjected to gel electrophoresis and western blot as described above.

The procedure was repeated with three times with three different samples for quantification.

### Assessment of sialic acid modifications in mouse brain homogenates

GPI anchors were isolated as previously described, with few modifications [88]. Briefly, 10% mouse brain homogenates were prepared with RIPA buffer plus protease inhibitor cocktail (Roche) and protein content was determined as described before. As sialic acids in N-glycans would interfere with the analysis of GPI-anchor modifications, samples containing 100 μg of total protein were first subjected to deglycosylation for 4 h at 37°C using the PNGase F kit (New England Biolabs) according to the manufacturer`s protocol. To only yield PrP^C^ and avoid presence of interfering IgGs in eluates, deglycosylated samples were subsequently used for immunoprecipitation using monoclonal antibody POM1 that had been covalently linked to beads following the instructions of the Pierce Co-Immunoprecipitation Kit (Thermo Scientific) before. Thy-1 was immunoprecipitated with the rat anti-mouse Thy-1 (MCA1474, Serotech). The eluted samples containing purified deglycosylated PrP^C^ were digested with 100 μg/ml proteinase K, at 37°C for 24 hours, resulting in GPI anchors attached to the terminal amino acid. The released GPIs were extracted with water-saturated butanol, washed with water 5 times and loaded onto C18 columns. GPIs were eluted under a gradient of propanol and water. The presence of GPIs was detected by ELISA. Maxisorb immunoplates were coated with 0.5 μg/ml concanavalin A (binds mannose) and blocked with 5% milk powder. Samples were added and any bound GPI was detected by the addition of the phosphatidylinositol-reactive mAb 5AB3-11, followed by a biotinylated anti-mouse IgM (Sigma), extravidin-alkaline phosphatase and 1mg/ml 4-nitrophenyl phosphate.

The presence of phosphatidylinositol in GPI anchors was identified using mAb (5AB3-11) and specific glycans were detected with biotinylated lectins. Isolated GPI anchors were bound to nitrocellulose membranes by dot blot and blocked with 5% milk powder. Samples were incubated with mAb 5AB3-11, biotinylated SNA (detects terminal sialic acid residues bound α-2,6 or α-2,3 to galactose), biotinylated concanavalin A (detects mannose) or biotinylated RCA I (detects terminal galactose) (Vector Labs). Bound lectins were visualised using extravidin peroxidase and enhanced chemiluminescence. The mAb was visualised by incubation with a horseradish peroxidase conjugated anti-murine-IgG and chemiluminescence.

### Primary neurons

For preparation of primary hippocampal neurons, we used postnatal P0-P2 mice. Briefly, pups were killed by decapitation, and after removing skull and meninges, the hippocampus was dissected and collected in 10mM glucose in PBS containing 0.5 mg/ml papain (Sigma-Aldrich) and 10μg/ml DNAse (Roche). Hippocampi of PrP^C^GPIThy-1 mice were collected individually, and the tails were used for genotyping. After 30 min incubation at 37°C, samples were washed 4 times with plating medium (MEM 1X (Gibco), 20mM glucose (Sigma), 10% Horse serum (PAA Laboratories) and 3% of NaHCO_3_ 7.5% (Gibco)) and carefully pipetted up and down several times in order to mince the tissue. Cells were plated in 6-well plates containing coverslips previously treated with 0.5 mg/ml of Poly-L-Lys and incubated at 37°C in a 5% CO_2_ cell culture incubator. After 4 hours, the media was changed to Neurobasal A medium (Gibco) containing 2% of B27 serum, Glutamax (Gibco) and penicillin/streptomycin (PAA Laboratories). Next day, AraC (Sigma-Aldrich) was added to kill proliferating cells. Half of the media was changed every three days.

### Confocal immunofluorescence microscopy of primary neurons

After seven days in culture, coverslips were washed in PBS (Sigma-Aldrich), fixed (4% paraformaldehyde in PBS, 15 min room temperature (RT)), washed (PBS) and permeabilized with PBS containing 0.1% bovine serum albumin (BSA) and 0.3% Triton-X-100 for 1h at RT. Cells were then incubated with primary antibody (POM1 antibody at 1:250 and anti-tau antibody (Synaptic Systems, 1:500) diluted in PBS/0.1% BSA) for 1h at RT, washed (PBS), incubated with secondary antibody (donkey anti-mouse antibody AlexaFluor 488 and anti-guinea pig AlexaFluor 555 (Invitrogen), diluted in PBS/0.1% BSA, 1 h at RT) and washed again with PBS. DAPI (Roche) was added to the last wash and samples were then mounted with Fluoromount G media (SouthernBiotech). Consecutive Z-stacks (between 30–35 Z-stacks per picture) were taken with Leica Laser Scanner Confocal Microscope TCS SP5 (Leica), and the reconstructed 3D images were further processed with the IMARIS Software to quantify colocalization. For this, 12 pictures for the controls (from 5 animals) and 7 pictures for PrP^C^GPIThy-1 (from 5 animals) were analyzed. Out of every picture, ten regions of interest (ROI) were selected, and the total intensity of PrP and tau within these ROIs was measured. The ratio of PrP/tau intensity of 10 ROIs was then added to make a total mean. Total mean of all the pictures for WTPrP^C^ and the ones from PrP^C^GPIThy-1 neurons were then added and subjected to statistical analysis (unpaired Student's t-Test).

### DRM isolation

DRMs were isolated as previously described [29]. Briefly, about 0.2 mg of frontal cortex was homogenized in 10 vol. of homogenization buffer (10mM Tris-HCl pH 8.2, 0.02% sodium azide and 0.32M sucrose) on ice. After centrifugation at 500xg for 5 min to pellet nuclei and cell debris, the supernatant was further centrifuged for 40 min at 18,000xg to obtain a membrane-enriched fraction. The amount of protein from this fraction was quantified, and 1–2 mg of protein were diluted with 2x detergent buffer (10mM Tris-HCl pH 8.2, 1% Brij 96 (Sigma-Aldrich), 1% sodium deoxycholate (Sigma-Aldrich)) that had previously been mixed at least for 16h. Samples were then incubated for 30 min at 4°C and mixed at a ratio of 1:1 with 80% sucrose in the detergent buffer. 35% of sucrose (8 ml) and 5% of sucrose (1 ml) in detergent buffer were layered on top. After 18 h of centrifugation at 200,000xg, 12 fractions were taken and subjected to electrophoresis and western blot as previously described. Primary antibodies for DMR characterization used in the western blot were Flotillin (1:1,000; Cell Signaling Technologies) and calnexin (1:1,000; BD Transduction). For DRM isolation of samples depleted of myelin, a modification of the protocol from Chen *et al*. was used [31]. Briefly, samples were homogenized with ISB buffer (10mM HEPES/KOH pH 7.6; 200mM sucrose, 50mM K acetate, 1mM Mg acetate, 1mM EGTA, 1mM DTT and protease inhibitors) and centrifuged at 5000xg for 5 min. The resulting supernatant was further centrifuged at 22.000xg for 60 min to obtain a pellet with a membrane enriched fraction. This pellet was resuspended in ½ of the ISB buffer starting volume and centrifuged at 20.000xg for 60 min again. The resulting pellet was resuspended in 1ml ISB buffer and layered on a top of a 0.83M sucrose cushion in ISB buffer, and further centrifuged at 75.000xg for 35 min. After centrifugation, a myelin sheath band was visible at the border of the 0.83M sucrose. All the sample under the myelin layer was further diluted to 0.2M sucrose and centrifuged 30.000xg for 40 min. The resulting pellet was resuspended in 200 μl of ISB buffer without sucrose, the amount of protein quantified, and equal amounts of protein were further incubated either with 2X detergent (1% Brij 96/1% sodium deoxycholate in ISB buffer without sucrose) for 30 min at 4°C or with Brij 98 (Sigma-Aldrich) for 5 min at 37°C. After incubation, samples were mixed at a ratio of 1:1 with 80% sucrose in ISB buffer and proceeded with the sucrose gradient and ultracentrifugation as described above.

### Proteinase K (PK) digestion

Frontal cortex brain samples (n = 3 for WTPrP^C^; n = 4 for PrP^C^GPIThy-1) were homogenized 1:10 in RIPA buffer and digested with 20 μgrs/ml of PK (Roche) at 37°C for 1 h. The reaction was stopped as before. All samples were subjected to electrophoresis and western blot analysis.

### Cell culture, transfection, and co-cultivation

Cells were cultivated and transfected as described [89]. The human SH-SY5Y cell line is a human neuroblastoma cell line (DSMZ number ACC 209). Co-cultivation experiments were done as described previously [40, 90]. In brief, SH-SY5Y cells were grown on glass cover slips and transfected with Lipofectamine (Invitrogen). 2 h after transfection cover slips were transferred into dishes containing a 90% confluent cell layer of either N2a (immortalized neuroblastoma cell line, ATCC No. Ccl 131) or chronically infected ScN2a cells (established by infecting N2a cells with an enriched preparation of prions isolated from the brains of mice infected with RML prions [91]). After 16 h of co-cultivation, apoptotic cell death was analyzed (see below).

### Apoptosis assay

16 h after co-cultivation, SH-SY5Y cells were fixed on glass cover slips with 3.7% paraformaldehyde for 20 min, washed and permeabilized with 0.2% Triton X-100 in PBS for 10 min at room temperature. Fixed cells were incubated with an anti-active caspase 3 antibodies overnight at 4°C, followed by incubation with the fluorescently labeled secondary antibody Alexa Fluor 555 for one hour at room temperature. Cells were then mounted onto glass slides and examined by fluorescence microscopy using a Zeiss Axiovert 200M microscope (Carl Zeiss). The numbers of cells positive for activated caspase-3 out of at least 1,000 transfected cells were determined in a blinded manner. All quantifications were based on at least three independent experiments.

### Statistical analysis

IBM SPSS Statistics 22 and GraphPad Prism 5 statistic software programs were used in the statistical analysis. To assess differences between Kaplan-Meier survival curves, the Breslow test was used. For comparison between the groups in western blots and neuronal countings, unpaired Student's *t-*test was used. Statistical significance was considered when *p*-values were as follows: *p < 0.05, **p < 0.005, ***p < 0.001, ****p<0.0001. The exact *p* value is also given.

## Supporting information

S1 Fig Biochemical characterization of PrP^C^GPIThy-1 L27 and L16 mouse lines. (A) Bar chart of PrP^C^ mRNA levels measured by RT-qPCR. PrP^C^GPIThy-1 L16 (n = 6) presents a doubled amount of PrP mRNA levels compared to WTPrP^C^ (n = 7). (B) Representative western blots showing PrP^C^GPIThy-1 protein expression in different organs in the two transgenic mice lines compared to WTPrP^C^. (B: brain; Cb: cerebellum; SC: spinal cord; Sp: spleen; M: muscle). POM1 antibody was used to detect PrP.
(TIF)

S2 Fig No differences in lipid subdomain localization are observed between PrP^C^GPIThy-1 and WTPrP^C^. (A) DRMs isolation of WTPrP^C^ and PrP^C^GPIThy-1 mouse brain (n = 3 for each genotype) with a mixture of 0.5% Brij96 and 0.5% sodium deoxycholate. After extraction at 4°C and overnight centrifugation on a sucrose density gradient, twelve fractions were loaded on a gel. Flotillin is used as a marker for DRMs, whereas calnexin, a chaperone resident in the ER, is used as a marker of non-DRM fractions. Note that there no difference was found between controls and transgenic mice in the PrP solubilization pattern (quantifications are shown below). (B) Under the same conditions as in (A), Thy-1 stays in the DRMs (mainly in fractions 2 and 3). (C) Because myelin could impair the proper solubilization of the DRMs, in another set of experiments we depleted the samples from myelin and used the same procedure as in (A). Again, no differences in the solubilization pattern were seen by incubating with 0.5% Brij 96 and 0.5% sodium deoxycholate. With another detergent (Brij 98 at 37°C), although the isoform solubilization pattern differs between WTPrP^C^ and PrP^C^GPIThy-1, no differences in the fraction distribution were observed.
(TIF)

S3 Fig Isolated GPI-anchors from WTPrP^C^ present a sialic acid, which is absent in Thy-1. Dot blot analysis of PrP^C^ and Thy-1 GPI-anchors from WT mouse brain and PrP knock-out mice (Prnp^0/0^ mice). Phosphatidylinositol (PI), mannose (man) and sialic acid (sial. acid) were detected as described in the methods section. Note that the amounts of PI and mannose are similar between Thy-1 and WTPrP^C^, whereas sialic acid is only present in WTPrP^C^.
(TIF)

S4 Fig Relative mRNA amounts of WTPrP^C^ and PrP^C^GPIThy-1 L150 and biochemical characterization of PrP^C^GPIThy-1 L159. (A) Bar chart showing relative amounts of PrP^C^ mRNA extracted from brains of WTPrP^C^ and PrP^C^GPIThy-1 L150 mice showing no differences. WTPrP^C^ is set to 1. (B) Representative western blot of total brain homogenates from PrP^C^GPIThy-1L159 and quantification of the signal showing that these animals present around 50% of the transgene compared to amounts of PrP in WTPrP^C^ mice (n = 3 for each genotype; **p = 0.0011).
(TIF)

S5 Fig 22L prions also lead to a significant delay to terminal disease in PrP^C^GPIThy-1 L150. Kaplan-Meier survival curve of WTPrP^C^ and PrP^C^GPIThy-1 L150 mice inoculated with 22L prions. PrP^C^GPIThy-1 L150 mice (n = 5; black line) reached terminal disease and were sacrificed at day 156 ± 3 dpi, compared to 144 dpi for WTPrP^C^ (n = 5; grey line); Log Rank (Mantel-Cox) **p<0.003).
(TIF)

S6 Fig PrP^C^GPIThy-1 L16 inoculated either with RML or 22L presents with delay to terminal disease and different neuropathology. (A) Kaplan-Meier survival curve of PrP^C^GPIThy-1 L16 mice infected either with RML or 22L prions. Note the substantial delay to terminal disease for the transgenic mice (RML: 400 ± 56 dpi (n = 10); 22L: 200 ± 23 dpi (n = 5); black line) compared to WTPrP^C^ mice (RML: 157 ± 6 dpi (n = 8); 22L: 144 ± 1 dpi (n = 5); grey line; Log Rank (Mantel-Cox) RML: ****p<0.0001; 22L: **p = 0.003). (B) Neuropathological analysis of terminally diseased PrP^C^GPIThy-1 L16 mice infected with RML. In PrP^C^GPIThy-1 L16 brains a general decrease in spongiosis is observed with HE staining. Gliosis is also decreased as observed with antibodies against astrocytes (GFAP) and microglia (Iba1). (C) Representative blot of WTPrP^C^ and PrP^C^GPIThy-1 L16 brain homogenates infected with RML prions and digested with PK. Decreased amounts of PK-resistant PrP^Sc^ were observed for PrP^C^GPIThy-1 L16 brain homogenates, despite of the two-fold expression of the transgene (shown in S1A Fig).
(TIF)

S7 Fig Expression of the PrP^C^GPIThy-1 transgene is not changed with aging and PrP^C^GPIThy-1 L150 mice show relatively increased PrP shedding after RML infection. (A) Western blot analysis of PrP expression in PrP^C^GPIThy-1 L150 mice at 20 weeks (20W, n = 3) and 40 weeks of age (40W, n = 4). No changes in expression were observed between the two groups (bar chart shows the mean of PrP relative intensity related to actin used as a loading control; WTPrP^C^ is set to 100%). (B) (i) Representative western blot showing expression of shed PrP in terminally RML-infected mice (n = 4) compared with non-infected PrP^C^GPIThy-1 mice (n = 4). Note that shed PrP increases about 3-fold in RML-infected animals. Bar chart shows relative intensity of shed PrP related to the total amount of PrP (shown in the re-probed blot in (ii); PrP^C^GPIThy-1 in uninfected mice is set to 100%). (ii) Representative western blot showing that the amounts of total PrP do not change between RML-infected (n = 4) and uninfected PrP^C^GPIThy-1 mice (n = 4). The bar chart shows relative intensity of PrP developed with POM1 related to actin used as a loading control. All error bars are SEM.
(TIF)

S8 Fig Shedding of PrP is also decreased in PrP^C^GPIThy-1 L159. (A) Representative blot of total brain homogenates from WTPrP^C^ and PrP^C^GPIThy-1 L159 mice detected with the antibody against shed PrP. (B) Although this line of transgenic mice expresses *per se* less PrP^C^GPThy-1 levels than WTPrP^C^ mice (as shown in S3 Fig), when the corresponding signal of shed PrP is referred to the signal of total PrP, there is a significant decrease (*p = 0.015) in shed PrP in PrP^C^GPIThy-1 L159 brains (n = 3) compared to WTPrP^C^ (n = 3).
(TIF)

S1 Table Mouse lines used in the experiments. (DOCX)
