# Supplementary figures and images for: GPI-anchor signal sequence influences PrPC sorting, shedding and signalling, and impacts on different pathomechanistic aspects of prion disease in mice
Source: PLoS Pathog. 2019 Jan 4;15(1):e1007520. doi: 10.1371/journal.ppat.1007520 (PMC6334958; doi:10.1371/journal.ppat.1007520)

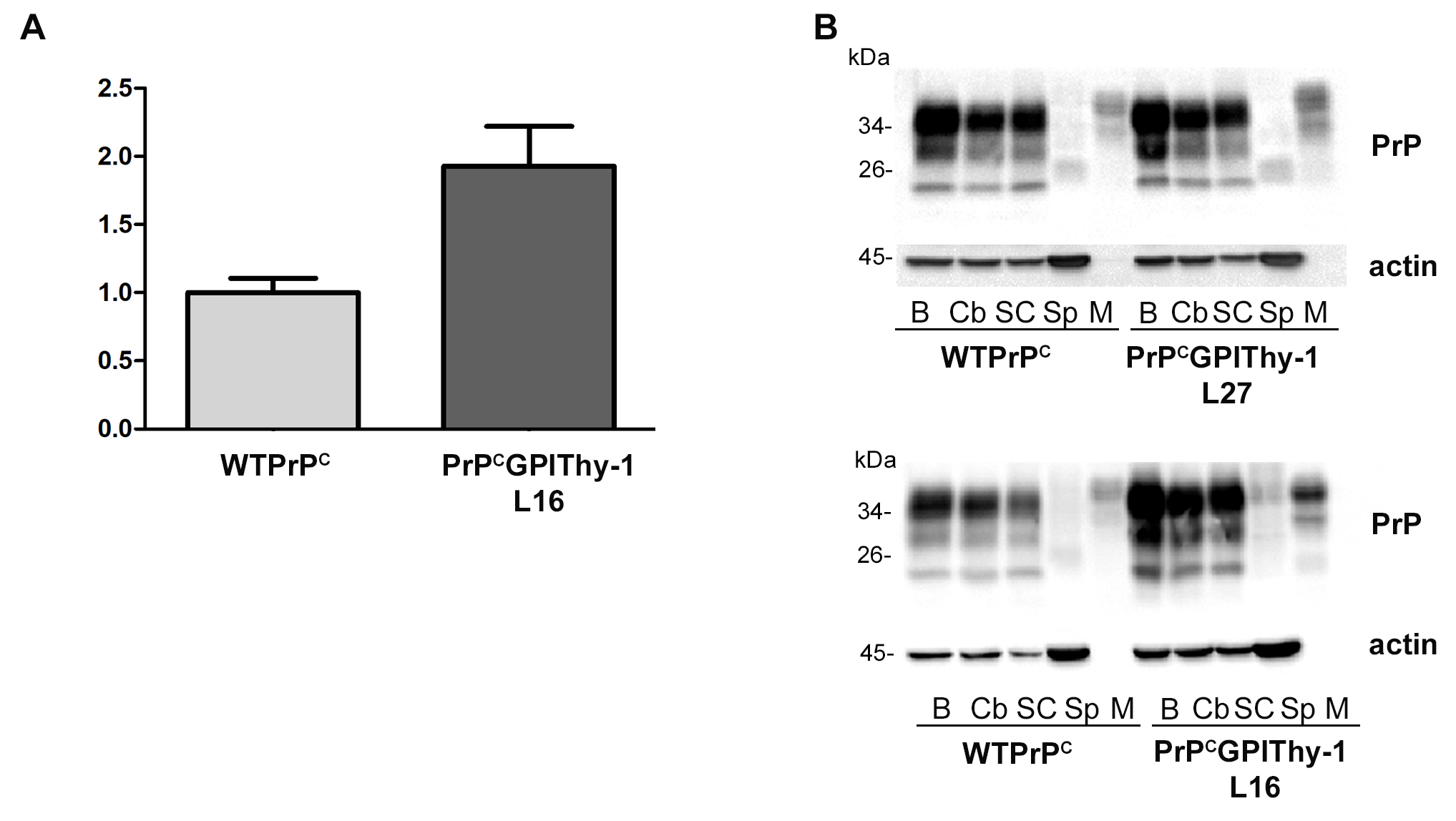

Supplement: S1 Fig — (A) Bar chart of PrPC mRNA levels measured by RT-qPCR. PrPCGPIThy-1 L16 (n = 6) presents a doubled amount of PrP mRNA levels compared to WTPrPC (n = 7). (B) Representative western blots showing PrPCGPIThy-1 protein expression in different organs in the two transgenic mice lines compared to WTPrPC. (B: brain; Cb: cerebellum; SC: spinal cord; Sp: spleen; M: muscle). POM1 antibody was used to detect PrP. (TIF) [file ppat.1007520.s001.tif]

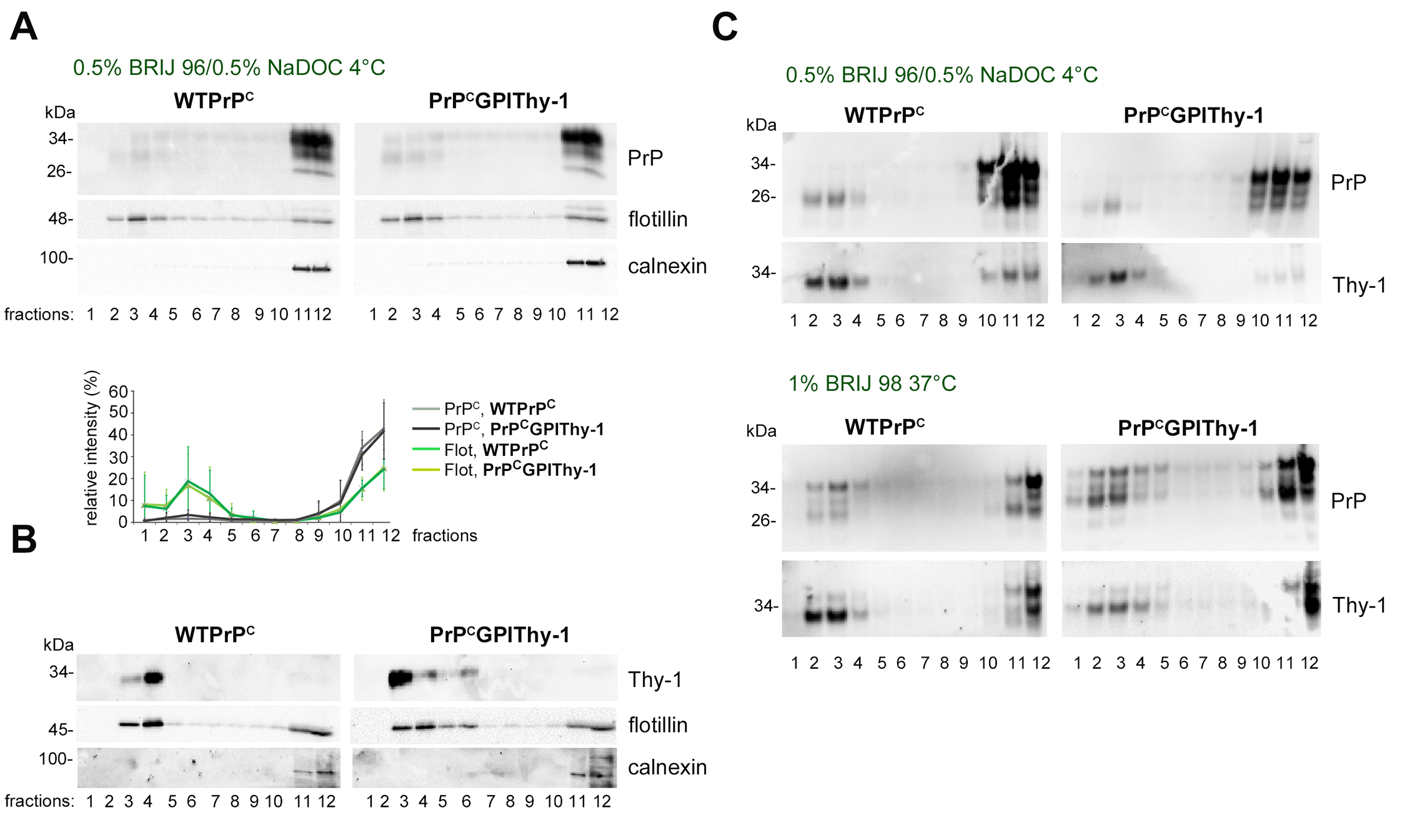

Supplement: S2 Fig — (A) DRMs isolation of WTPrPC and PrPCGPIThy-1 mouse brain (n = 3 for each genotype) with a mixture of 0.5% Brij96 and 0.5% sodium deoxycholate. After extraction at 4°C and overnight centrifugation on a sucrose density gradient, twelve fractions were loaded on a gel. Flotillin is used as a marker for DRMs, whereas calnexin, a chaperone resident in the ER, is used as a marker of non-DRM fractions. Note that there no difference was found between controls and transgenic mice in the PrP solubilization pattern (quantifications are shown below). (B) Under the same conditions as in (A), Thy-1 stays in the DRMs (mainly in fractions 2 and 3). (C) Because myelin could impair the proper solubilization of the DRMs, in another set of experiments we depleted the samples from myelin and used the same procedure as in (A). Again, no differences in the solubilization pattern were seen by incubating with 0.5% Brij 96 and 0.5% sodium deoxycholate. With another detergent (Brij 98 at 37°C), although the isoform solubilization pattern differs between WTPrPC and PrPCGPIThy-1, no differences in the fraction distribution were observed. (TIF) [file ppat.1007520.s002.tif]

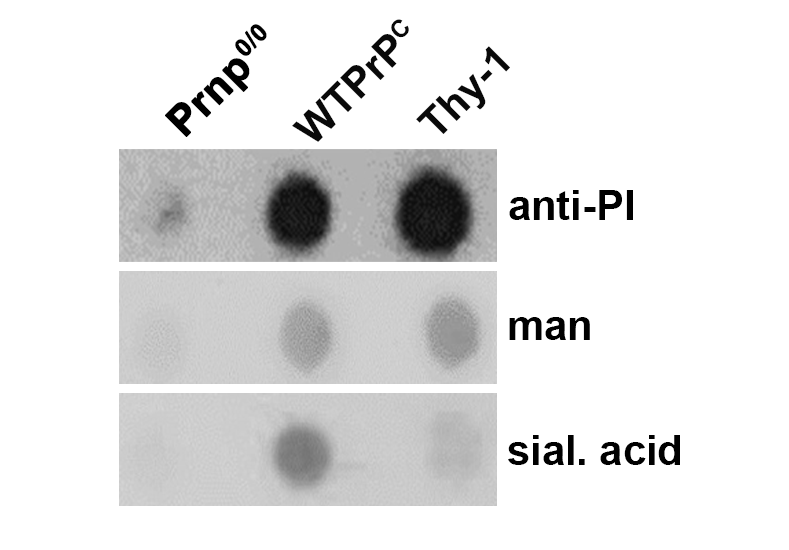

Supplement: S3 Fig — Dot blot analysis of PrPC and Thy-1 GPI-anchors from WT mouse brain and PrP knock-out mice (Prnp0/0 mice). Phosphatidylinositol (PI), mannose (man) and sialic acid (sial. acid) were detected as described in the methods section. Note that the amounts of PI and mannose are similar between Thy-1 and WTPrPC, whereas sialic acid is only present in WTPrPC. (TIF) [file ppat.1007520.s003.tif]

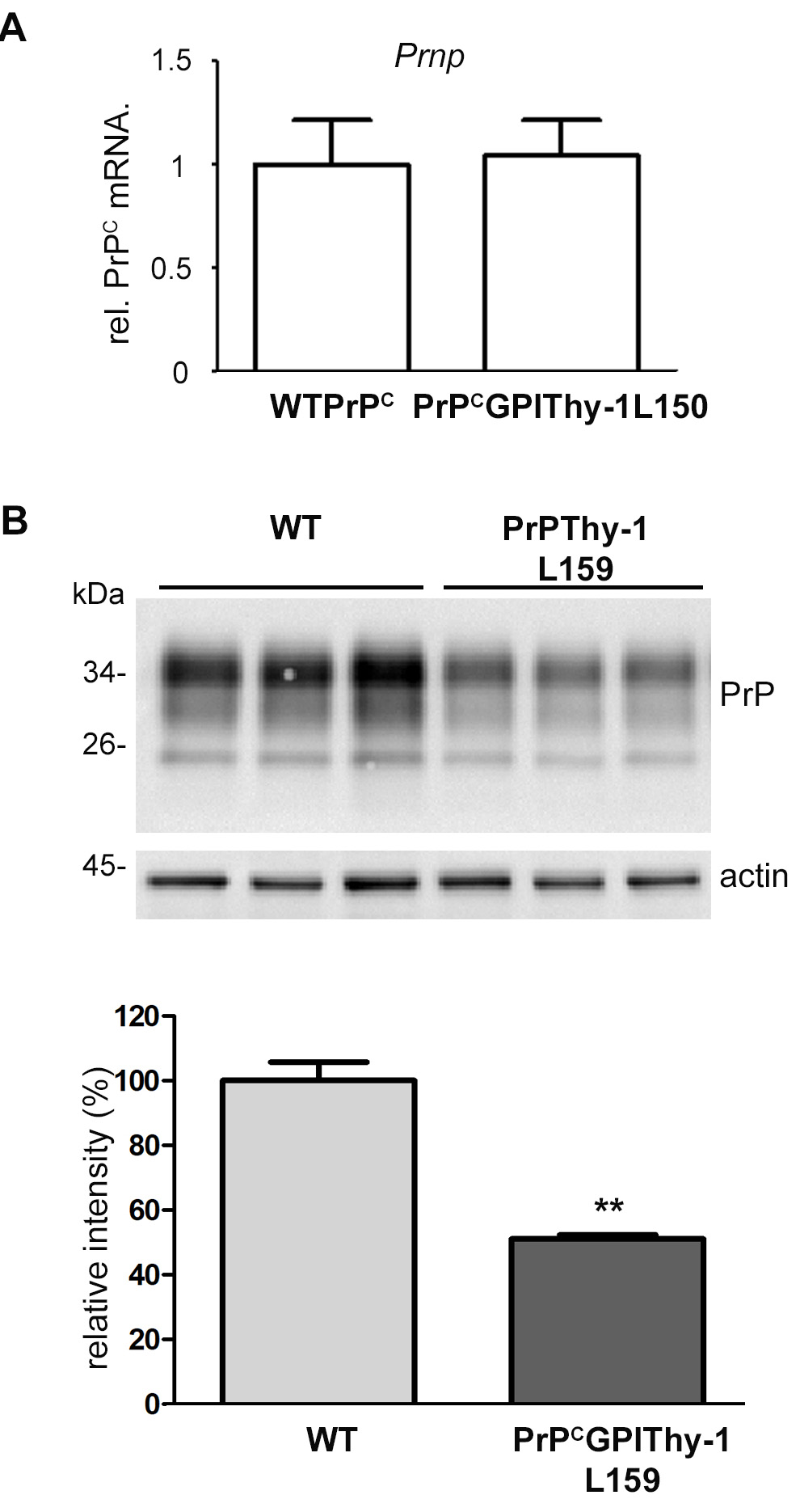

Supplement: S4 Fig — (A) Bar chart showing relative amounts of PrPC mRNA extracted from brains of WTPrPC and PrPCGPIThy-1 L150 mice showing no differences. WTPrPC is set to 1. (B) Representative western blot of total brain homogenates from PrPCGPIThy-1L159 and quantification of the signal showing that these animals present around 50% of the transgene compared to amounts of PrP in WTPrPC mice (n = 3 for each genotype; **p = 0.0011). (TIF) [file ppat.1007520.s004.tif]

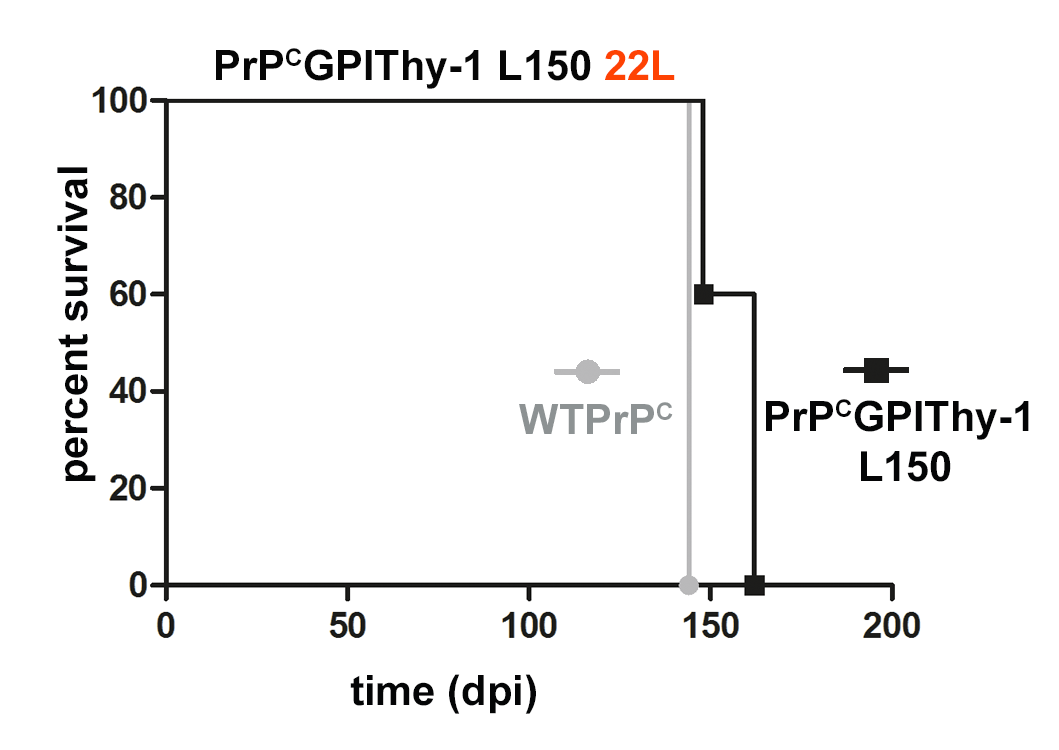

Supplement: S5 Fig — Kaplan-Meier survival curve of WTPrPC and PrPCGPIThy-1 L150 mice inoculated with 22L prions. PrPCGPIThy-1 L150 mice (n = 5; black line) reached terminal disease and were sacrificed at day 156 ± 3 dpi, compared to 144 dpi for WTPrPC (n = 5; grey line); Log Rank (Mantel-Cox) **p<0.003). (TIF) [file ppat.1007520.s005.tif]

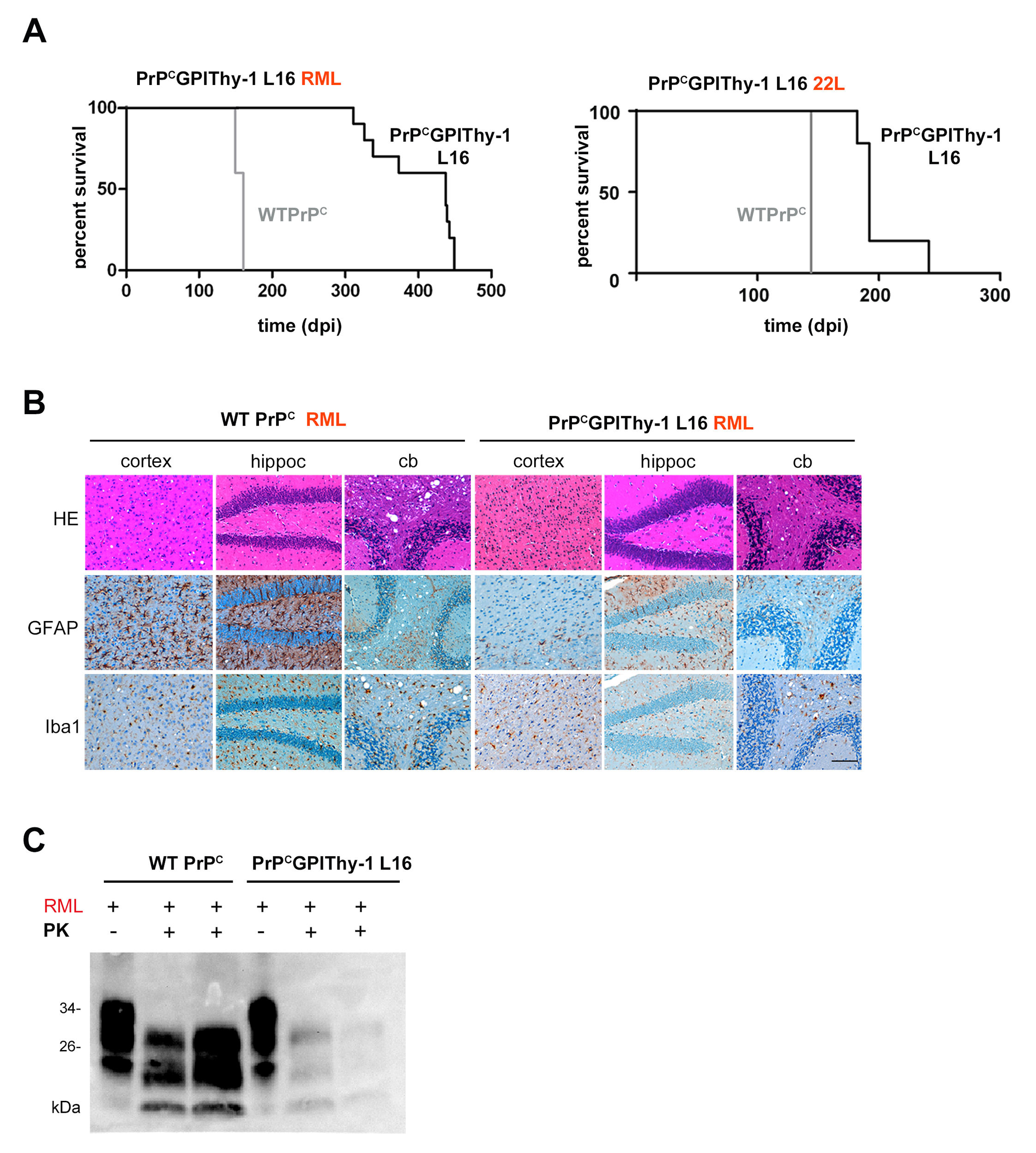

Supplement: S6 Fig — (A) Kaplan-Meier survival curve of PrPCGPIThy-1 L16 mice infected either with RML or 22L prions. Note the substantial delay to terminal disease for the transgenic mice (RML: 400 ± 56 dpi (n = 10); 22L: 200 ± 23 dpi (n = 5); black line) compared to WTPrPC mice (RML: 157 ± 6 dpi (n = 8); 22L: 144 ± 1 dpi (n = 5); grey line; Log Rank (Mantel-Cox) RML: ****p<0.0001; 22L: **p = 0.003). (B) Neuropathological analysis of terminally diseased PrPCGPIThy-1 L16 mice infected with RML. In PrPCGPIThy-1 L16 brains a general decrease in spongiosis is observed with HE staining. Gliosis is also decreased as observed with antibodies against astrocytes (GFAP) and microglia (Iba1). (C) Representative blot of WTPrPC and PrPCGPIThy-1 L16 brain homogenates infected with RML prions and digested with PK. Decreased amounts of PK-resistant PrPSc were observed for PrPCGPIThy-1 L16 brain homogenates, despite of the two-fold expression of the transgene (shown in S1A Fig). (TIF) [file ppat.1007520.s006.tif]

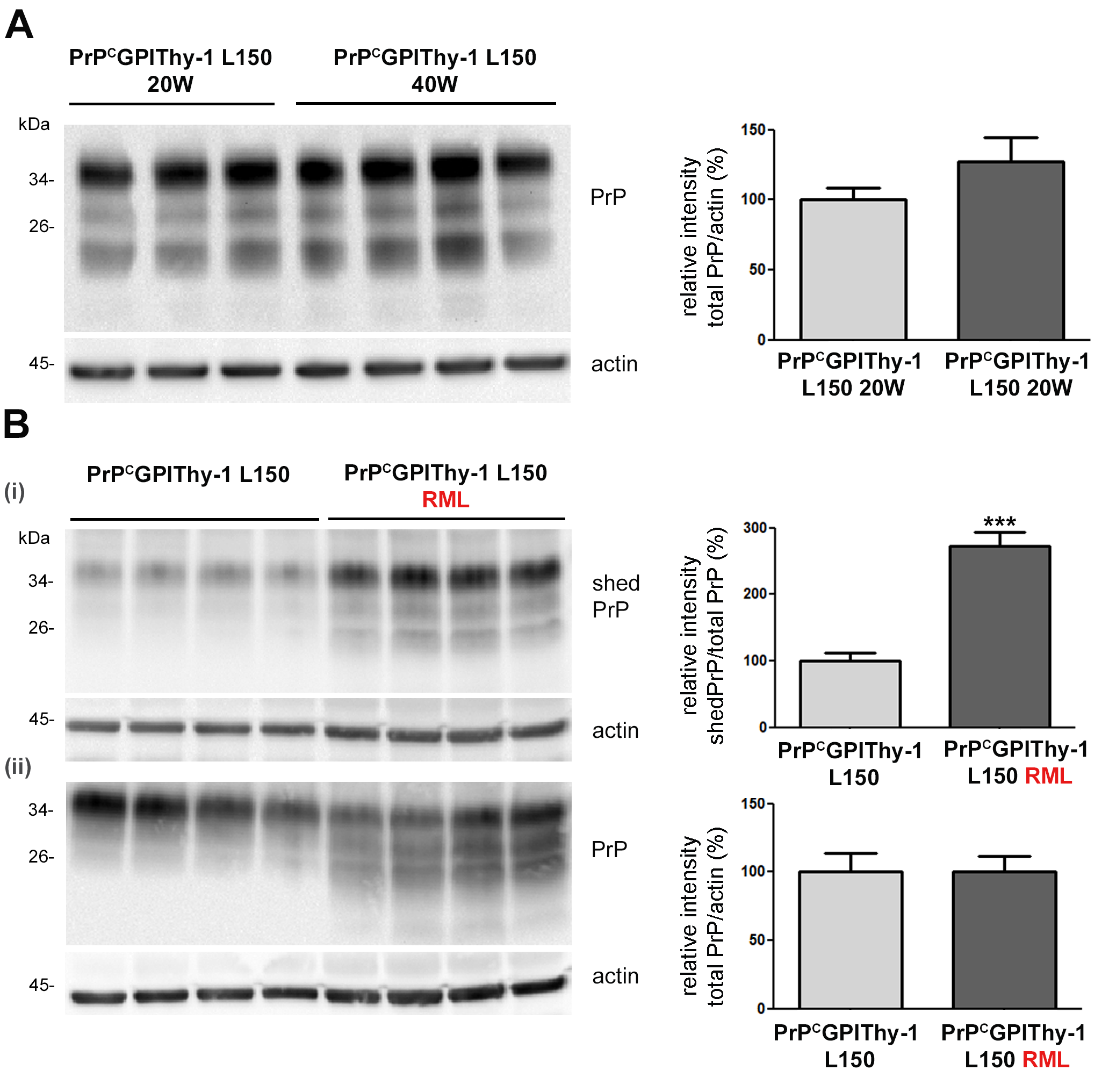

Supplement: S7 Fig — (A) Western blot analysis of PrP expression in PrPCGPIThy-1 L150 mice at 20 weeks (20W, n = 3) and 40 weeks of age (40W, n = 4). No changes in expression were observed between the two groups (bar chart shows the mean of PrP relative intensity related to actin used as a loading control; WTPrPC is set to 100%). (B) (i) Representative western blot showing expression of shed PrP in terminally RML-infected mice (n = 4) compared with non-infected PrPCGPIThy-1 mice (n = 4). Note that shed PrP increases about 3-fold in RML-infected animals. Bar chart shows relative intensity of shed PrP related to the total amount of PrP (shown in the re-probed blot in (ii); PrPCGPIThy-1 in uninfected mice is set to 100%). (ii) Representative western blot showing that the amounts of total PrP do not change between RML-infected (n = 4) and uninfected PrPCGPIThy-1 mice (n = 4). The bar chart shows relative intensity of PrP developed with POM1 related to actin used as a loading control. All error bars are SEM. (TIF) [file ppat.1007520.s007.tif]

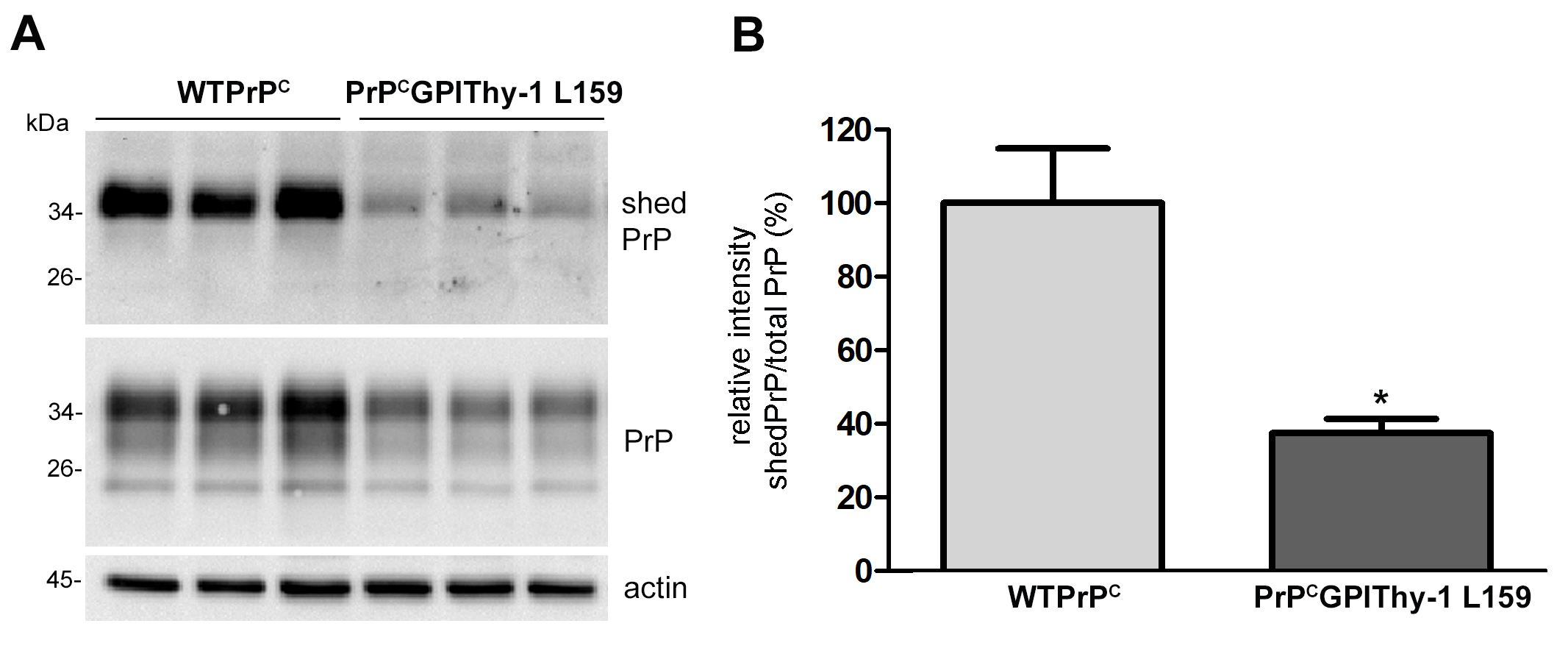

Supplement: S8 Fig — (A) Representative blot of total brain homogenates from WTPrPC and PrPCGPIThy-1 L159 mice detected with the antibody against shed PrP. (B) Although this line of transgenic mice expresses per se less PrPCGPThy-1 levels than WTPrPC mice (as shown in S3 Fig), when the corresponding signal of shed PrP is referred to the signal of total PrP, there is a significant decrease (*p = 0.015) in shed PrP in PrPCGPIThy-1 L159 brains (n = 3) compared to WTPrPC (n = 3). (TIF) [file ppat.1007520.s008.tif]
