# Supplementary material for: GPI-anchor signal sequence influences PrPC sorting, shedding and signalling, and impacts on different pathomechanistic aspects of prion disease in mice
Source: PLoS Pathog. 2019 Jan 4;15(1):e1007520. doi: 10.1371/journal.ppat.1007520 (PMC6334958; doi:10.1371/journal.ppat.1007520)
Supplement: S1 Table — (DOCX) [file ppat.1007520.s009.docx]

| **MICE** | **EXPERIMENT** | **FIGURES** |
| --- | --- | --- |
| **PrP^C^GPIThy-1 L27**  **(3F4 tag)** | WB analysis  qPCR  TX 114 assay  DRMs isolation  Primary neurons ICC  Isolation and characterization of GPI anchors | 1C, S1B Fig.  1B  1D  S2 Fig  2B, 2C  1E, 1F |
| **PrP^C^GPIThy-1 L16**  **(3F4 tag)** | WB analysis  qPCR  Inoculation with RML and 22L prions  Neuropathological assessment of RML inoculation  PK-resistance characterization | S1B Fig  S1A Fig  S6A Fig  S6B Fig  S6C Fig |
| **PrP^C^GPIThy-1 L150** | WB analysis  qPCR  Inoculation with RML prions  Neuropathological assessment of RML inoculation  PK-resistance characterization  Shedding analysis  ERK and p38 expression  Inoculation with 22L prions | 3A  S4 Fig  3B  3C, 3D  4A, 4B,4C, 4D  5A, 5B, 5C, 5D, 5F, S7A, B Fig  6A, 6B  S5 Fig |
| **PrP^C^GPIThy-1 L159** | WB analysis  Shedding analysis | S4 Fig  S8 Fig |
